# Regulation of Expression of Extracellular Matrix Proteins by Differential Target Multiplexed Spinal Cord Stimulation (SCS) and Traditional Low-Rate SCS in a Rat Nerve Injury Model

**DOI:** 10.3390/biology12040537

**Published:** 2023-03-31

**Authors:** Dana M. Tilley, Ricardo Vallejo, Francesco Vetri, David C. Platt, David L. Cedeño

**Affiliations:** 1 Research and Development, SGX Medical, Bloomington, IL 61704, USA; 2 Neuroscience Program, Illinois Wesleyan University, Bloomington, IL 61701, USA; 3 Pain Management, National Spine and Pain Centers, Bloomington, IL 61704, USA

**Keywords:** neuropathic pain, spinal cord stimulation (SCS), extracellular matrix (ECM), spared nerve injury (SNI), differential target multiplexed programming (DTMP), phosphorylation, proteomic, matrisome

## Abstract

**Simple Summary:**

The extracellular matrix comprises an important collection of proteins that aids in the body’s response to pain. Evaluating the effect of electrical neuromodulation treatments on expression levels in neural tissues associated with neuropathic pain is important for improving such treatments. Here, we analyzed extracellular matrix proteins in spinal cords of a rodent model of neuropathic pain. We evaluated two spinal cord stimulation (SCS) therapies, one based on differential target multiplexed programming (DTMP) and another based on a conventional low-rate program (LR-SCS), for their ability to reverse the effects associated with the pain model. Of the 186 proteins identified as extracellular-matrix-related, DTMP reversed expression levels of 83% of them back to levels seen in uninjured animals, whereas LR-SCS reversed 67%. Protein phosphorylation is indicative of activation/deactivation of signaling pathways. Phosphorylation can occur at multiple locations on a protein and multiple times as well. There were 883 unique phosphorylated states found among 93 ECM-related proteins. Relative to the pain model, DTMP had a reversal effect on 76% versus 58% by LR-SCS on phosphorylated proteins. This study helps up to better understand pain pathways and also highlights a more robust reversal by DTMP relative to LR-SCS.

**Abstract:**

There is limited research on the association between the extracellular matrix (ECM) and chronic neuropathic pain. The objective of this study was twofold. Firstly, we aimed to assess changes in expression levels and the phosphorylation of ECM-related proteins due to the spared nerve injury (SNI) model of neuropathic pain. Secondly, two modalities of spinal cord stimulation (SCS) were compared for their ability to reverse the changes induced by the pain model back toward normal, non-injury levels. We identified 186 proteins as ECM-related and as having significant changes in protein expression among at least one of the four experimental groups. Of the two SCS treatments, the differential target multiplexed programming (DTMP) approach reversed expression levels of 83% of proteins affected by the pain model back to levels seen in uninjured animals, whereas a low-rate (LR-SCS) approach reversed 67%. There were 93 ECM-related proteins identified in the phosphoproteomic dataset, having a combined 883 phosphorylated isoforms. DTMP back-regulated 76% of phosphoproteins affected by the pain model back toward levels found in uninjured animals, whereas LR-SCS back-regulated 58%. This study expands our knowledge of ECM-related proteins responding to a neuropathic pain model as well as providing a better perspective on the mechanism of action of SCS therapy.

## 1. Introduction

Chronic pain is the result of a summation of multiple processes including neuroinflammation, regulation of ion transport, and glial activation. The disruption of pathways associated with these processes can lead to the perturbation of neuron–glial homeostasis, dysregulation of ion trafficking, depolarization of neurons, and ultimately the perception of pain. A structure that has recently gained traction as an intrinsic factor in pain regulation is the extracellular matrix (ECM). The ECM is a network of proteins and other molecules surrounding, supporting, and giving structure to cells. It enables the release of proteins that regulate inflammatory cascades, membrane channels, and cell–cell connections in neuron–glial and interglial networks. A search in Pubmed for “ECM and pain” showed that before 1990 there were only 9 papers published, by 2000 there were 84, and by 2010 there were 381, growing exponentially to 1350 papers by 2020. Technological advancements, including transcriptomic and proteomic analysis, have expanded our ability to understand the complex nature of the ECM and its role in pain.

The combination of ECM and ECM-related proteins, such as intracellular networks and signaling proteins, constitute what is known as the matrisome. This is a complex environment that is important for responding to an injury, the healing process, and reestablishing damaged pathways [1]. The perineural net is the portion of the ECM supporting synapses, which is critical in neuroplasticity. Neuroplasticity refers to the modification of neuronal connections due to changes in the environment and is involved in establishing chronic neuropathic pain [2]. The perineural net is established and maintained by both neurons and glial cells and the neuron–glial interactions that regulate pain perception. Glial cells have been shown to migrate along the ECM to target destinations in a process partially dependent on ECM stiffness [3] and signaling molecules [4]. Following an injury, matrix metalloproteases (MMPs) are released and facilitate deconstruction of the ECM. Reactive gliosis, specifically microglia and astrocyte activation, has been observed along with ECM deconstruction induced by MMPs due to the spared nerve injury (SNI) model of neuropathic pain [5]. MMP inhibitors reversed glial activation and restored neuron–glial transporter expression, although microglia still showed signs of activation, as opposed to astrocytes which appeared to become inactive. This evidence suggests that the role of ECM pain-related regulation is differentially effective against astrocytes and microglial cells.

The ECM is composed of a variety of interwoven filament proteins anchored to cell-membrane proteins. Although ECM proteins are primarily considered to be extracellular, there are also intracellular ECM-related proteins that act as either anchors for integrins to maintain localization in the membrane or proteins bound to integrins, such that changes to the ECM can modulate intracellular activities directly (see Figure S1 for a general overview). ECM structural proteins, such as collagens and laminins, strengthen cell–cell connections and maintain proximities between cells as well as facilitating cell migration along the ECM [6]. This is especially important within the neuron–glial network in the formation and strengthening of synapses between neurons.

Presynaptic membrane proteins such as neurexin, and postsynaptic ones such as neuroligin, are known to interact in the synaptic cleft to maintain a close neuron–neuron association. In addition to synapse formation, they also play a role in anchoring synaptic ion channel proteins, which is important in neuron excitability [7,8,9,10]. In the context of pain, MMPs are considered inflammatory proteins that cleave other ECM proteins. Protein cleavage by MMPs in pain-related processes contribute to the release of proteins that activate inflammatory cascades and to the weakening of the cell network connectivity, which results in the disruption of neuron–glial homeostasis [11]. Tajerian et al. observed altered geometrical motifs and a reduced fiber diameter in the ECM following induction of a model of peripheral chronic pain [12].

Structural changes within the ECM can affect signaling pathways primarily through integrins anchored to cell membranes and attached to the ECM. Signaling molecules, such as growth factors, associated with the ECM can be cleaved to bind receptors for pathway activation or be held in close proximity to their receptors, so that changes to the ECM allow presentation of signaling proteins to their receptors. For example, transforming growth factor beta (TGFβ), which has been shown to be embedded within the ECM, can modulate cell response as well as ECM composition [13,14]. ECM stiffness also plays a role in cell migration, proliferation, and survival [15]. Glial cells secrete components of the ECM that have been shown to be modulated following nerve injury [16,17]. From a pathophysiological perspective, peripheral nerve injury promotes an immune and inflammatory response in the central nervous system, which involves the activation of glial cells, which in turn affects the homeostatic balance as a result of changes in neuron–glial interactions. These are manifested as phenotypic changes that result in the maladaptive transformation of structural cellular components, including the ECM, which ultimately lead to changes in neural plasticity.

Spinal cord stimulation (SCS) is an electrical neuromodulation therapy for treating chronic neuropathic pain. It has been used for decades and is still undergoing investigation regarding its mechanisms of action to optimize outcomes. One such SCS treatment involves a differential target multiplexed programming (DTMP) approach in which multiple pulsed electrical signals of varying parameters regulate the effect of neurons and glial cells in a painful condition. Parameters such as pulse width, frequency, amplitude, latency period, and active vs. passive recharge can have a differential effect on the neuron–glial environment. Our group reported the modulation of genes related to the neuron–glial network following induction of the SNI model as well as reversed expression changes as a result of treatment with DTMP [16,17]. Proteomic analyses allowed the study of the effect of SCS on pain-related pathways using the SNI model of neuropathic pain. We also reported on the effects of SCS on ion transport [18], the mTOR pathway [19], and pathways related to neuroinflammation [20]. In a previous study, we evaluated proteomic expression changes in stimulated spinal cord tissue using a conventional clinically relevant SCS program. The study showed that approximately 1/3 of the SCS-modulated proteins were ECM-related [21].

This study evaluates the matrisome following induction of the SNI pain model using proteomic analyses. Additionally, we studied the effectiveness of SCS using either DTMP or a conventional low-rate SCS (LR-SCS) program in reversing changes to the ECM induced by the pain model. Phosphorylation changes of matrisome proteins were also evaluated, as phosphorylation can lead to the activation or inactivation of pathways that are not likely to produce significant protein expression changes of their non-phosphorylated state.

## 2. Materials and Methods

### 2.1. Rodent Model of Neuropathic Pain and SCS Therapies

Experimental details are provided elsewhere [22]. Briefly, procedures were approved by the Institutional Animal Care and Use Committee at Illinois Wesleyan University. Male rats (Sprague-Dawley, Envigo RMS, Indianapolis, IN, USA) weighing in the 275–315 g range were maintained in a temperature and humidity-controlled room with a 12 h light/dark cycle. Animals were housed in individual cages with food and water ad libitum. Experimental groups consisted of untreated animals (No-SCS, n = 10), SCS-treated animals (DTMP, LR-SCS, n = 10 each), or uninjured animals (No-SNI, n = 10), which did not receive either the SNI model or SCS. A cylindrical quadrupolar SCS lead (1 mm Pt/Ir electrodes, 0.62 mm diameter, Evergreen Medical, St. Paul, MN, USA) was implanted at the L1-L2 vertebral level in the No-SCS, DTMP, and LR-SCS animals followed by implementation of the SNI model as described previously [22]. Five days later, DTMP and LR-SCS animals were stimulated for forty-eight hours continuously. LR-SCS consisted of charge-balanced single pulses at a rate of 50 Hz and 150 µs pulse width (PW). DTMP consisted of charge-balanced pulsed signals with components at 50 Hz (150 µs PW) and 1200 Hz (50 µs PW) distributed over the four electrodes of the lead [22,23]. Signal intensities were in the 0.03–0.10 mA range, corresponding to about 70% of the motor threshold, and were not adjusted throughout the stimulation period. No-SCS animals were connected to the SCS device for forty-eight hours, but the signal intensity was set to zero. All groups were assessed in parallel by a researcher blinded to the assignments for the extent of mechanical hypersensitivity before surgical intervention, before starting the stimulation period and right before the end of the stimulation period.

### 2.2. Protein Isolation and Quantification and Proteomic Analysis

Animals were euthanized after the final assessment, followed by dissection of the ipsilateral dorsal quadrant of the stimulated spinal cord. Tissues were washed with cold saline, snap-frozen and stored at −80 °C. Samples from 4 responders to treatment (improvement in mechanical hypersensitivity corresponding to at least 30% of the pre-SNI measurement) were used in this study, while 4 further samples were used for transcriptomics analyses previously reported [22]. Proteomic analyses were conducted by scientists in Cell Signaling Technology (Danvers, MA, USA) blinded to the experimental group assignments. To separate proteins out, homogenized tissues were suspended in a urea (9M) buffer free of ionic detergents and enriched with protease inhibitors. After determination of the amount of total protein content in the samples, aliquots were trypsinized and cysteine residues alkylated using standard methods [24]. Tryptic peptides from the four biological specimens were pooled for each experimental group and labeled using a tandem mass tag (TMT) system designed for simultaneous identification, quantification, and comparison of proteins between experimental groups [25]. Combined tagged peptides were combined and fractionated via reverse column liquid chromatography (LC) using a linear gradient of acetonitrile in 0.125% formic acid (280 nL/min for 150 min). Combinations of eluted fractions were subjected to LC/Tandem Mass Spectrometry (TMS) using multi-notch MS3 methodology. Analyses were conducted using optimized protocols (Cell Signaling Technology, Danvers, MA, USA). Mass spectra were evaluated using SEQUEST and the Core platform from Harvard University [26], and the Uniprot rat protein database was used to search for proteins. Results from the search were filtered on precursor ions (±5 ppm mass accuracy) and further filtered to a 1% protein level false discovery rate (FDR). Fold changes for evaluating the effect of the pain model (No-SCS vs. No-SNI), and treatments (DTMP vs. No-SCS and LR-SCS vs. No-SCS) was obtained by ratioing the normalized spectral intensities (log_2_ scale) of tagged peptides that were uniquely assigned to each protein. Two-tailed *t*-tests were used to assess the statistical significance (*p* < 0.05) of fold changes for each protein identified and quantified. StringDB v11.0 [27] was used to build protein–protein interaction networks for significantly modulated proteins. Significantly enriched biological processes modulated by the pain model and either SCS treatment were obtained using gene ontology enrichment analysis (GOEA) and the Panther database [28]. Secondary confirmation of the involvement of target proteins in ECM-related pathways was obtained using the Kyoto Encyclopedia of Genes and Genome (KEGG) [29].

### 2.3. Phosphoproteomic Analysis

Tryptic peptides were purified using reversed-phase solid-phase extraction and phospho-enriched with iron-based magnetic beads (PTMScan^®^ Fe-IMAC, Cell Signaling Technology, Danvers, MA, USA) via immobilized metal affinity chromatography (IMAC) [30]. Immobilized phosphopeptides were eluted with basic pH buffer after washing out unbound peptides. Phosphopeptides were purified using reversed-phase LC and injected for LC/TMS analyses using standard techniques (Cell Signaling Technology, Danvers, MA, USA). Technical assays were run for each pool of biological samples in duplicate due to the limited amount of protein available after the whole proteomic analysis. Fold changes were obtained from the spectral intensities of phosphoproteins filtered as described above. Although reliable, comparisons could not be assessed for statistical significance. The relative variability of fold changes was assessed via their coefficient of variation (CV), calculated as the square root of the sum of the square of the individual CVs of the ratioed values, as previously reported [20]. Phosphoproteins that experienced at least a 10% change in expression value in the No-SCS group relative to the No-SNI were deemed to be affected by the pain model. Those affected by the pain model and in which expression levels were reversed by at least 10% with DTMP or LR-SCS treatment relative to untreated (No-SCS) and the expression in uninjured animals (No-SNI) were deemed to be back-regulated.

## 3. Results

Behavioral studies on these animals, presented elsewhere [22], confirmed sensitization due to the SNI pain model (paw withdrawal threshold, PWT, of 24.6 ± 2.4% for No-SCS relative to pre-injury, baseline) which was significantly reversed by both DTMP (PWT of 62.9 ± 8.9% relative to baseline) and LR-SCS (PWT of 37.6 ± 6.0% relative to baseline). Animals selected for proteomic analyses had mean PWT (relative to baseline) of 27.0 ± 8.5% for No-SCS, 68.6 ± 12.8% for DTMP, and 40.1 ± 6.8% for LR-SCS. Mean signal intensities (and corresponding charge density) used in these animals were 0.06 ± 0.02 mA (0.44 ± 0.15 nC/s) for LR-SCS and 0.07 ± 0.03 mA (0.51 ± 0.26 nC/s, low-rate signal) and 0.04 ± 0.01 mA (2.6 ± 0.5 nC/s, high-rate signal) for DTMP, which were representative of the overall animal population in each group, which were 0.06 ± 0.02 mA (0.47 ± 0.17 nC/s) for LR-SCS, and 0.07 ± 0.03 mA (0.56 ± 0.24 nC/s, low-rate signal) and 0.06 ± 0.03 mA (3.6 ± 1.5 nC/s, high-rate signal) for DTMP.

The proteomic analysis identified 7192 proteins in the excised spinal cord tissue of these animals. Of those, 1451 proteins were significantly affected by DTMP relative to No-SCS animals, versus 705 proteins significantly affected by LR-SCS. There were 186 proteins identified that were involved in the ECM; 177 identified by Panther and 9 identified in literature searches. Based on the bioinformatic tools, these proteins can be classified as being related to cell structure, protein signaling cascades, and cell junction organization and assembly. This includes the formation of neuron synapses, localization and anchoring of glial cells, and neuronal signaling via both ion channels as well as extra and intracellular cascades. Many of these proteins have multiple functions and therefore have overlapping roles.

### 3.1. Proteomic Expression of ECM-Related Proteins

#### 3.1.1. Modulated Expression of ECM Structural Organization Proteins

**Figure 1:**
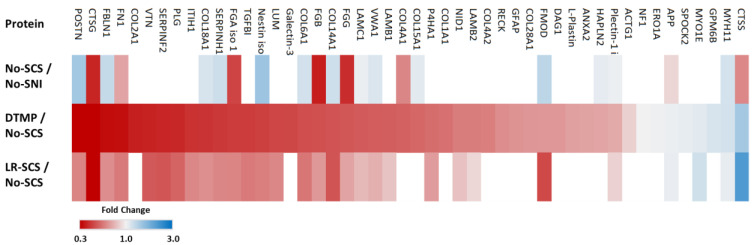
Fold-change (FC) heatmap of the ECM structural proteins (n = 48). Red (decrease) and blue (increase) represent significantly changed expression levels, and white stands for no significant change.

ECM structural organization proteins, of which 48 were found in the dataset, consist largely of proteins that form fibers or long chains such as collagens and laminins (Figure 1, Table S1). Expression levels of actin, ACTG1, were unaffected by No-SCS or LR-SCS and decreased with DTMP. The Plectin, PLECTIN-1, level was increased by the pain model and decreased by DTMP and LR-SCS. These are intracellular structural proteins that serve as anchors for the ECM. Some glial-specific intracellular structural proteins such as the glial fibrillary acidic protein (GFAP), downregulated by DTMP, and nestin (NES), upregulated by the pain model and downregulated by both DTMP and LR-SCS, have been co-observed with changes in ECM composition. Other extracellular structural proteins like collagens (nine identified in dataset), laminins (three identified in dataset), hyaluronan linking proteins (two identified in dataset), and proteoglycans (nine identified in dataset) were also affected. Of the nine collagens, expression levels of five (COL4A1, COL6A1, COL14A1, COL15A1, COL18A1) were significantly affected by the pain model. Expression levels of all collagens were decreased with DTMP, with four of the five affected by pain being significantly reversed by DTMP toward levels found in uninjured animals. In contrast, LR-SCS reversed three of them with no further effect observed on the others. Similarly, expression levels of all laminins (LAMB1, LAMB2, LAMC1) were significantly decreased with DTMP. Expression of LAMC1, affected by the pain model was back-regulated by both DTMP and LR-SCS, whereas expression levels of the other two were decreased with DTMP and LR-SCS treatment. Levels of two hyaluronan linking proteins were increased by pain and reversed by DTMP with only one of two, HAPLN2, being reversed by LR-SCS. Out of the nine proteoglycans, DTMP decreased expression levels in six of them, while it increased expression levels in the other three. There were three proteoglycans that were affected by the pain model, with all three being reversed by both DTMP and LR-SCS.

#### 3.1.2. Modulated Expression of Adhesion and Cell Junction ECM Proteins

Adhesion of cells to other cells or to the ECM allows for temporary cell–cell communication and may be a precursor for establishing cell junctions. Indeed, cell adhesion and cell junction proteins are heavily interconnected. We found 28 proteins significantly modulated by DTMP that were identified as cell–cell adhesion proteins and 112 as cell junction proteins. Of the 28 cell adhesion proteins, DTMP increased the expression levels of 7 relative to No-SCS. All seven showed no significant change in either No-SCS or LR-SCS groups. Upregulated proteins involved neuron–neuron adhesion proteins such as NLGN3 and NRXN1, neuron–cell adhesion proteins such as neural cell adhesion molecule L1, NCAM-L1, and general cell–cell adhesion proteins such as CADM1 and CTNND2. DTMP decreased expression levels of the remaining 21 proteins relative to No-SCS. Out of these, 10 were only affected by DTMP treatment (including catenin proteins CTNNA1 and CTNND1), 6 were significantly upregulated by the pain model and back-regulated by DTMP (including cytoskeletal proteins vinculin, and FLNA), and the remaining 5 were downregulated by the pain model and further downregulated by DTMP (including ECM connectivity proteins FG (B and G) and FN1). In comparison, LR-SCS only had a significant effect on 9 out of the 28 cell adhesion proteins, all of which had reduced expression levels, with 5 of them being back-regulated relative to the No-SCS group.

**Figure 2:**
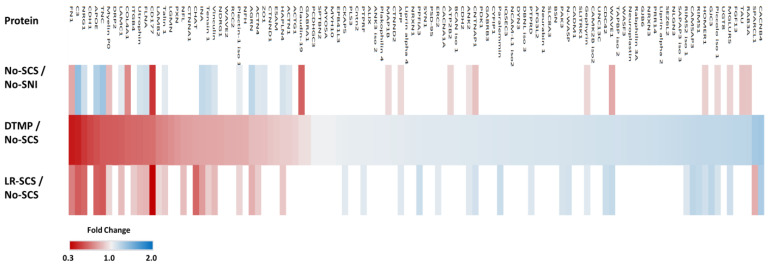
Fold-change (FC) heatmap of the ECM cell junction proteins (n = 112). Red (decrease) and blue (increase) represent significantly changed expression levels, and white stands for no significant change.

Cell junction proteins are involved in the formation and modulation of the cell junctions. There were 112 proteins (Figure 2, Table S2) identified as cell junction related proteins that modulate the cell junction, including some G-coupled protein membrane receptors, such as mGluR5 which helps to regulate the breakdown of the ECM. The expression level of mGluR5 was significantly decreased by the pain model and back-regulated by DTMP and LR-SCS. Gap junction proteins, such as GJB6, which was upregulated by DTMP only, or the glial structural protein GFAP, downregulated by DTMP only, are examples of cell-junction-related proteins.

#### 3.1.3. Modulated Expression of Cell Signaling ECM Proteins

**Figure 3:**
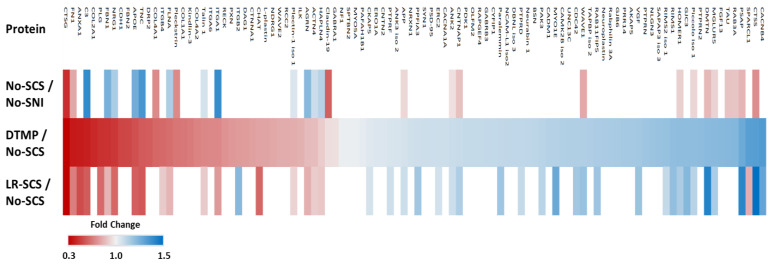
Fold-change (FC) heatmap of signaling proteins associated with the ECM (n = 102). Red (decrease) and blue (increase) represent significantly changed expression levels, and white stands for no significant change.

We identified 102 proteins involved in cell signaling cascades (Figure 3, Table S3), with 73 of them classified as signal transduction proteins. These mainly consist of signaling molecules anchored within the ECM that can be cleaved to interact with their receptors, such as the fibroblast growth factor 13 (FGF13), which was upregulated by DTMP. They also consist of proteases that release small peptide fragments from the ECM called matrikines or matricryptins, and proteases such as cathepsins (CTSS, CTSG) responsible for ECM degradation and the release of bioactive signaling peptides. Both DTMP and LR-SCS back-regulated CTSS, which was upregulated by the pain model. DTMP and LR-SCS, however, further decreased the expression level of CTSG, which was decreased by the pain model.

Integrins are ECM-related proteins found in cell membranes of all cell types that facilitate cell–cell communication and the activation of signaling pathways. Four integrins primarily linked to glial cells that have been reported to be involved in inflammation were identified in the proteomic data (ITGA1, ITGA6, ITGB2, and ITGB4) [31,32,33]. Only ITGA1 was significantly affected (upregulated) by the pain model and was back-regulated by both DTMP and LR-SCS. DTMP significantly downregulated the other three while LR-SCS downregulated one, had no effect on another one, and increased expression levels of ITGB2. Other proteins that regulate cell signaling via phosphorylation or dephosphorylation are kinases and phosphatases, respectively. There were five kinases (AKAP5, CAMK2B, ILK, PAK3, and PDK1) identified, though the pain model did not alter their expression levels significantly. DTMP increased expression levels of all of them except for ILK, which was decreased. The LR-SCS effect on these kinases was limited to PAK3, which was similarly altered by DTMP. There were four subunits of one phosphatase, each with modulated expression levels. The protein tyrosine phosphatase receptor (PTPR) is a protein made of multiple subunits of which four (PTPRF, PTPRN, PTPRN2, and PTPRD) were found in the proteomic data. No significant change was observed for any of these subunits due to the pain model, but DTMP increased expression levels of all. LR-SCS increased expression levels of two of them.

### 3.2. Modulation of Phosphoproteomic States of ECM-Related Proteins

Due to the well-established nature of phosphoregulation on cellular activity and on/off switching for pathway activation, we evaluated changes in the phosphorylation of ECM related proteins. As phosphorylation is a means by which neural cells can rapidly activate or inhibit neuropathic pain pathways, the identification of regulatory targets modulated by SCS treatment is important. Phosphoproteomic analyses evaluated all 186 ECM related proteins, leading to the identification of 93 phosphorylated ECM proteins encompassing 883 phosphorylated states, indicative of large effects on pathway activation associated with post-translational modifications. The pain model affected expression levels (by at least 10%) in 738 (83.6%) of these phosphorylated isoforms. DTMP was able to back-regulate 559 (75.7%) of these towards expression levels found in uninjured animals, whereas LR-SCS was able to back-regulate 428 of them (58.0%).

#### 3.2.1. Changes in Phosphorylation States of Structural ECM Proteins

**Figure 4:**
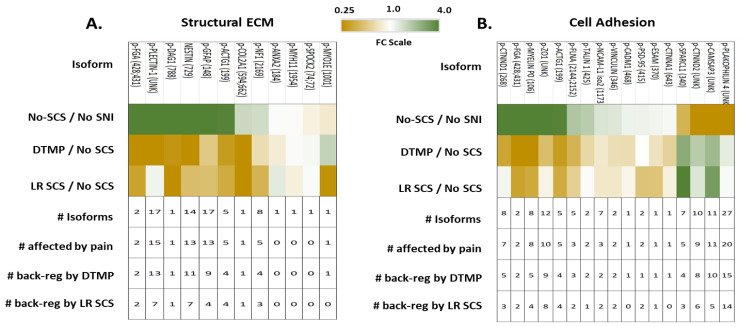
Heat maps with expression level fold changes (FC) of selected phosphoproteins isoforms associated with (**A**) structure of the ECM, (**B**) cell adhesion at the ECM. Only the isoforms with the largest expression change due to the pain model are shown for each phosphoprotein. Values in the table below the heat map indicate the number of isoforms identified, how many were affected by the pain model and how many of these were back-regulated (back-reg) by DTMP or LR SCS.

Out of the 48 structural ECM proteins identified in the proteomic analyses, 12 were phosphorylated, with 69 isoforms identified. The pain model affected expression levels of 56 (81%) of these isoforms by at least 10% relative to uninjured animals, with DTMP and LR-SCS back-regulating 46 (82%) and 29 (52%) of them, respectively. Figure 4A (Table S4) summarizes the findings and illustrates a fold-change heat map of the 12 isoforms most affected by the pain model. Intracellular structural proteins associated with the ECM appeared more regulated via phosphorylation than extracellular structural components as exemplified by ACTG1, PLECTIN-1 and glial-specific proteins GFAP and NES. These proteins were differentially affected by the pain model as well as DTMP and LR-SCS. We identified five p-ACTG1 isoforms affected by the pain model. DTMP and LR-SCS back-regulated expression levels of four of these toward levels found in uninjured animals. The pain model affected expression levels of 15 of 17 p-PLECTIN-1 isoforms identified. DTMP and LR-SCS back-regulated expression levels of 13 and 7 of them, respectively. There were 17 phosphorylated isoforms of GFAP, with 13 affected by the pain model, of which 9 and 4 were back-regulated by DTMP and LR-SCS, respectively. We also found 14 p-NES isoforms, with 13 affected by the pain model within 11 and 7 of those back-regulated by DTMP and LR-SCS, respectively.

Phosphorylation of proteins comprising extracellular structural components of the ECM is not extensive, indicating little to no phospho-regulation of ECM structural proteins outside the cell membrane. However, washout of phosphorylation residues due to our extraction and isolation method cannot be excluded. Of the nine collagens identified in the proteomic data, only one was found to be phosphorylated (p-COL2A1), which was affected by the pain model and back-regulated by both DTMP and LR-SCS. No phosphorylated laminins or hyaluronan and proteoglycan linking proteins (HAPLN) were identified. Of the nine proteoglycans in the proteomic dataset, only SPOCK2, BCAN, and DAG1 were found to be phosphorylated, with a total of five p-isoforms. Of these five proteoglycan isoforms, four were affected by the pain model, with DTMP back-regulating one and LR-SCS back-regulating two. Additionally, no phosphorylated fibronectins were identified and only one phosphorylated fibrinogen (p-FGA) was found, with two p-isoforms that were affected by the pain model, which were back-regulated by both DTMP and LR-SCS towards levels found in uninjured animals.

#### 3.2.2. Changes in Phosphorylation States of Adhesion and Cell Junction ECM Proteins

Out of the 93 ECM proteins identified to be phosphorylated, 17 are cell adhesion proteins (out of 28 identified in the proteomic database) and 70 are cell junction proteins (out of 112 identified in the proteomic database). The 17 cell adhesion phosphoproteins have a total of 111 isoforms, of which 92 (83%) were affected by the pain model, with 75 (82%) being back-regulated by DTMP and 59 (64%) by LR-SCS. Figure 4B (Table S5) summarizes the findings and presents a heat map with the 17 isoforms most affected by the pain model. Neither neurexins nor neuroligins were identified in the phosphoproteomic database. Of the cell adhesion molecules (CAMs), seven p-NCAM isoforms were observed, with three of them affected by the pain model and two of them back-regulated by both DTMP and LR-SCS. CADM1 had one phosphorylated isoform which was affected by the pain model and back-regulated by DTMP but not LR-SCS. Of the three catenin proteins found in the proteomic data, all three yielded nineteen phosphorylated isoforms, of which seventeen were affected by the pain model, and DTMP was shown to back-regulate expression levels of fifteen, whereas LR-SCS back-regulated nine. Of the other proteins mentioned in the proteomics section, only vinculin (VCL) and FLNA showed to be phosphorylated. VCL had two isoforms that were affected by the pain model and back-regulated by both DTMP and LR-SCS. The filamin protein, FLNA, had five isoforms, with three affected by the pain model, and all tree back-regulated by DTMP, whereas two were back-regulated by LR-SCS.

**Figure 5:**
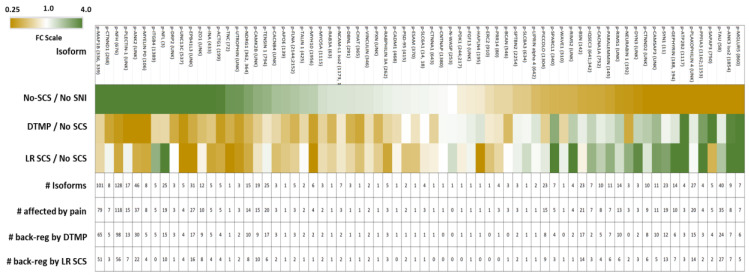
Heat map with expression level fold changes (FC) of selected phosphoproteins isoforms associated with cell junction at the ECM. Isoforms with the largest expression change due to the pain model are shown for each phosphoprotein. Values in the table below the heat map indicate the number of isoforms identified, how many were affected by the pain model and how many of these were back-regulated (back-reg) by DTMP or LR SCS.

The 70 cell junction proteins identified were shown to have 790 p-isoforms with 664 (84%) affected by the pain model. DTMP back-regulated phosphorylation levels of 509 of these (77%) and LR-SCS back-regulated 390 (59%). Figure 5 (Table S6) summarizes the findings and presents a heat map with the isoforms most affected by the pain model for each identified phosphoprotein associated with cell adhesion. Of the G-coupled protein receptors identified via the GOEA as part of the ECM, only mGLUR5 was found to be phosphorylated. This glutamate receptor had seven affected isoforms, of which six were modulated by the pain model, with six back-regulated by DTMP and five by LR-SCS. Only the gap junction protein GJC3 was identified in the phosphoproteomic data, with three isoforms found and all three affected by the pain model, while two were back-regulated by DTMP with no effect observed by LR-SCS.

#### 3.2.3. Changes in Phosphorylation States of Cell Signaling ECM Proteins

**Figure 6:**
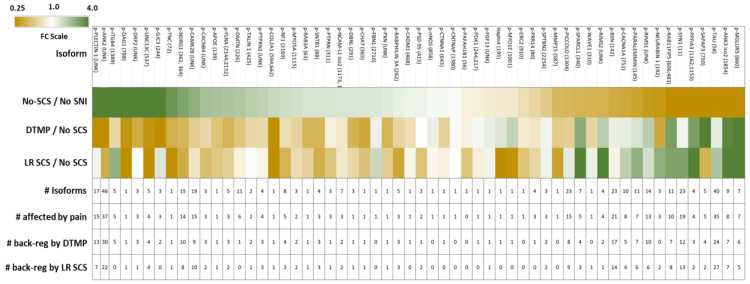
Heat map with expression level fold changes (FC) of selected ECM phosphoproteins isoforms associated with the cell signaling. Isoforms with the largest expression change due to the pain model are shown for each phosphoprotein. Values in the table below the heat map indicate the number of isoforms identified, how many were affected by the pain model and how many of these were back-regulated (back-reg) by DTMP or LR SCS.

The proteomic analysis identified 102 proteins, with 58 of them found to be phosphorylated. These proteins are mostly intracellular and transmembrane proteins connected to the ECM via integrins or anchoring proteins, with 29 classified as signal transduction proteins. The 58 phosphoproteins comprise 394 isoforms, of which 325 (82%) were strongly affected by the pain model, with DTMP reversing 237 (73%), and LR-SCS reversing 197 (61%). Figure 6 (Table S7) summarizes the findings and presents a heat map with the isoforms most affected by the pain model for each identified phosphoprotein. The only growth factor seen to be phosphorylated was FGF13, which has one isoform that was affected by the pain model and back-regulated by both DTMP and LR-SCS. There were 7 different receptors classified as ECM related proteins (CACNA1A, CACNB4, CNTNAP1, mGLUR5, ITGB4, NCAM-L1, and SLC6A1) of which 37 were phosphorylated isoforms. There were 30 isoforms affected by pain, of which 22 were back-regulated by DTMP and 14 by LR-SCS. Of the integrin proteins, only ITGB4 was found to be phosphorylated, having five different isoforms. All of them were upregulated by the pain model and back-regulated by DTMP, while LR-SCS did not back-regulate them. The kinases and phosphatases that were identified in the proteomic data did not appear to undergo extensive phosphorylation. Of the 5 kinases, only CAMK2B (19 isoforms with 15 affected by pain, 9 back-regulated by DTMP and 10 by LR-SCS) and PDK1 (1 isoform affected by the pain model and back-regulated by DTMP but not LR-SCS) were found to be phosphorylated. Of the four phosphatase PTPR protein subunits, only PTPRN (three isoforms affected by pain, one back-regulated by DTMP and LR-SCS) and PTPRN2 (four isoforms affected by SNI, three back-regulated by both DTMP and LR-SCS) underwent phosphorylation.

## 4. Discussion

The ECM is a dynamic cellular component that acts as a medium through which cell communication is filtered while responding to changes on environmental conditions resulting from processes such as inflammation [34]. This current work has identified more than one hundred ECM and ECM-related proteins and corresponding phosphorylated isoforms which were differentially affected by neuropathic pain model SCS treatments.

**Figure 7:**
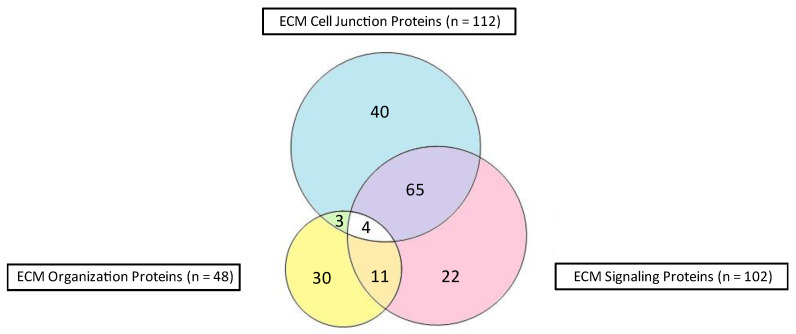
Venn diagram showing the overlap of different functions of the proteins of the matrisome identified in our dataset. Circle sizes are scaled based on the number of proteins and the number of shared proteins between the three groups.

Many ECM proteins have overlapping roles in structural support and organization, cell adhesion and junctions, and cell signaling (Figure 7). Of the three general classifications (structural, adhesion/junction, signaling) which the identified proteins have been sorted into, approximately thirty-eight percent (out of forty-eight) of the structural proteins were also co-identified as either adhesion or signaling proteins. Roughly 64% (out of 112) of the adhesion proteins and 78% (out of 102) of the signaling proteins were also identified as belonging to the other 2 groups. In total, four proteins were identified in all three groups.

Of the 48 identified ECM-related structural proteins, 22 were significantly affected by the SNI pain model, with 14 of these being upregulated. DTMP back-regulated levels of most of these except for two proteins (COL4A1 and CTSG). DTMP decreased expression levels of 39 of the 48 structural proteins. Three of the structural proteins with increased expression levels following DTMP were the chondroitin sulfate proteoglycans (CSPGs) brevican (BCAN), testican (SPOCK2), and neurocan (NCAN) which are primarily expressed by astrocytes. Recent work has shown that these CSPGs were degraded by activated microglia cells which facilitated the development of the SNI pain model [35]. Another study showed that the degradation of the ECM and loss of CSPGs is part of the inflammatory response, and that the return of CSPGs to the ECM facilitated the repair of the CNS environment [36]. Congruent with these reports, we observed a significant decrease of expression levels of NCAN due to the SNI model. A significant increase in expression levels of NCANs as well as BCAN and SPOCK2 was observed with DTMP treatment, though only NCAN was significantly increased following LR-SCS.

Both microglia and astrocytes become activated following a peripheral or central injury, leading to the development and maintenance of neuropathic pain [37]. Astrocytes play a key role in the production of ECM molecules in the inflamed and demyelinated spinal cord [38]. We found that the pain model increased expression levels of nestin (NES), both an astrocyte activation marker and a structural component of the ECM [39]. Following stimulation with DTMP, expression levels of nestin and the astrocyte activation marker GFAP (also involved in ECM modulation [40]) were decreased toward levels found in uninjured animals. In contrast, LR-SCS had no significant effect on GFAP, and although it significantly decreased levels of nestin, the reversal effect was only half as large as that of DTMP. Interestingly, we found that protein phosphorylation of structural ECM proteins was more extensive in those that are anchored in the cytosol than those located outside the cell. Besides the increase of nestin expression levels associated with the pain model, 13 out of the 14 p-nestin identified had expression levels elevated (21–732%) due the pain model. Phosphorylation of nestin has previously been associated with structural cell changes due to glial scar formation after CNS injury, particularly by integration into filament proteins together with other fibrillar proteins such as vimentin [41]. DTMP provided a more profound modulatory effect than LR-SCS by back-regulating eleven of these nestin phosphoisomers while LR-SCS back-regulated seven. Similarly, phosphorylation of GFAP, the archetypical astrocyte activation marker, has been previously found to be associated with pathological conditions of the CNS [42]. Our results indicate that the SNI pain model increased expression levels (by 20–540%) of 11 of the 17 p-GFAP isoforms identified, with DTMP providing back-regulation of 7 of these, versus LR-SCS, which only back-regulated 2 of them.

Actin (ACTG1) and plectin (PLEC) are intracellular structural proteins which act as anchoring points for ECM proteins [43] and are known to play a role in inflammation [44], similar to GFAP and NES in astrocytes. The expression level of PLEC was increased due to the pain model, while expression levels of both PLEC and ACTG1 were decreased by DTMP. LR-SCS also back-regulated PLEC but had no effect on ACTG1. ACTG1 and PLEC interact as main components of the cytoskeleton. Laminins have been shown as having an increased expression levels in an inflammatory model when GFAP levels were high [40], consistent with the increase we observed in the laminin protein, LAMC1, due to the SNI pain model. DTMP treatment decreased levels of both GFAP and all three laminin proteins, although LR-SCS only affected the laminins. Overall, DTMP reversed 12 of the 13 SNI-induced effects on structural protein expression versus LR-SCS, which reversed 10. Given that DTMP had a downregulating effect on 39 of the 48 identified ECM-related structural proteins, it is apparent that DTMP, and to a lesser extent LR-SCS, modulates expression and shifts the ECM environment toward a non-inflammatory phenotype.

Cell adhesion and cell junctions in the ECM are closely tied with cell signaling. Of the 112 cell junction proteins identified, 34 were affected by the pain model with 29 and 23 of those being back-regulated by DTMP and LR-SCS, respectively. Neuroinflammatory conditions are known to reduced expression levels of gap junction proteins, a subset of cell junction proteins [45]. DTMP induced an increase in expression levels of gap junction proteins GJB6 and GJC3 while LR-SCS only increased GJC3 levels. Attachment of glial cells at the site of pain-signaling neurons, leading to altered neuronal signaling, is governed by the ECM mainly via cell adhesion molecules (CAM). These proteins act as cell anchors and support signaling directly between cell types by maintaining cell–cell proximity. Of the 28 CAM-related proteins, 11 were affected by the SNI pain model, and expression levels of 6 were increased. Levels of all six were back-regulated by DTMP and five of them by LR-SCS toward levels found in injured animals. Of the other five proteins in which SNI decreased the expression level, DTMP further decreased expression levels of all five, whereas LR-SCS decreased levels of four with no change on pleckstrin. One CAM with previous precedence in the pain literature is NCAM-L1, with expression levels significantly increased by DTMP, although not affected by the pain model or LR-SCS. It has been shown to decrease astrocyte activity by inhibiting MAPK and microglia inflammatory processes by inhibiting TNFa and NO production [46]. Other CAM proteins reported in the pain literature are the neuron–neuron CAM proteins neurexin-1 (NRXN1) and neurolignin-3 (NLGN3). Expression levels of both of them were unaffected by the pain model but increased with DTMP treatment, while no effect was observed with LR-SCS. Levels of NRXN1 in neurons were shown to be decreased in an inflammatory pain model [47]. NLGN3 is known to play a role in forming or strengthening inhibitory synapses [48].

As mentioned earlier, the ECM is closely associated with cell–cell communication, and thus there was a lot of overlap between cell junction proteins and cell signaling proteins. Of the 102 ECM-related signaling proteins identified, 69 were also classified as ECM cell junction related proteins. There were 28 signaling proteins significantly affected by the pain model with 23 and 21 back-regulated by DTMP and LR-SCS, respectively. An involvement of ECM molecules such as integrins in signal transduction between glial cells as well as neurons due to inflammation has been observed [49]. Integrins act as anchors for other ECM proteins while intracellularly linked to signaling pathway proteins. They can lead to the formation or destruction of cell junctions to regulate cell–cell communications. We identified four integrin proteins, all of which had reduced expression levels with DTMP versus two by LR-SC, including ITGB4. Knockdown of ITGB4 has been shown to activate microglia and increase inflammation [50], while overexpression of ITGB4 promoted cell scattering and cell motility in combination with upregulation of vimentin expression, which is a sign of astrocyte activation [51]. Here we found that DTMP and, to a lesser extent, LR-SCS decreased expression levels of ITGB4, and previously we reported that DTMP was able to reduce vimentin expression [20]. Although the role of phosphorylation of ITGB4 in pain models has not been determined, it is quite interesting that all five p-ITGB4 identified in our database were upregulated by the pain model (12–807%), with DTMP back-reversing all of them while LR-SCS increased expression levels further.

Neuronal signaling and activity is significantly affected by shifts in concentration of ions, such as calcium. In the spinal dorsal horns, intracellular mGluR5 is primarily located on nuclear membranes, where activation leads to sustained Ca^2+^ responses. Nociceptive hypersensitivity induced by nerve injury has been shown to increase the expression of nuclear mGluR5 and decrease the cytoplasmic and plasma membrane expression. Moreover, spinal blockade of intracellular mGluR5 reduces neuropathic pain behaviors [52]. Here, we report a decrease in mGLUR5 expression due to the SNI model that was reversed with DTMP and LR-SCS. Although our results seem to diverge from the literature, it has to be noted that the difference in methodology and our inability to examine expression in specific cellular fractions might account for the differences. The role of phosphorylated mGLUR5 isoforms in neuropathic pain is not well established, but it may involve the alteration of synaptic plasticity as a result of enhancing localization of the receptor in the cell-surface facilitated by scaffolding proteins (such as Homer1) at the postsynaptic terminal [53]. Phosphorylation at the most distal part of the C-terminal cytoplasmic domain of mGLUR5, specifically at T1164 and S1167, facilitates its binding with Homer1 [54], while phosphorylation at S1126 has been demonstrated in an inflammatory pain model [55]. We identified 7 p-mGLUR5 isoforms, which were downregulated by the pain model (13–91%), all of them serine-phosphorylated at the cytoplasmic terminal domain. In total, six of them were back-regulated by DTMP (by 40% or more) towards expression levels found in uninjured animals, while LR-SCS back-regulated 5 of them (by 25% or more).

Our results confirm previously reported accounts on the important involvement of the ECM in neuropathic pain. Structural modifications of the ECM in perineural networks associated with dendritic modifications lead to maladaptive plastic changes linked to the chronification of pain [16]. Changes in neural plasticity are part of the central sensitization process which derives from neuroimmune and neuroinflammatory processes resulting from peripheral nerve injury. The involvement of microglial and astrocyte activation as a result of these is clearly linked to the reshaping of the ECM and concomitant changes in the intercellular interactions at the synapse, which ultimately affect cell signaling and neurotransmission [56]. The effects of astrocyte activation on expression levels of proteins in the ECM that are associated with this process is also confirmed by our analyses. Our recent work on the effect of SCS on key signaling neuroinflammatory pathways, namely the MAPK and mTOR pathways [19,20], also highlighted the influence of astrocyte activation. The identification of ECM signaling proteins that are also cell adhesion molecules implies that the ECM serves as a structural component by which signaling processes are modulated. The large changes in phosphorylated proteins and expression levels associated with the pain model is indicative of such a signaling modulation. The effects of DTMP treatment on expression levels of ECM proteins affected by the pain model in comparison with those exerted by LR-SCS are comparable to the effects observed in proteins associated with MAPK- and mTOR-mediated signaling and regulation of ion transport [18]. In all these cases, we have documented that DTMP has a more profound effect at regulating expression levels of affected proteins (and phosphoproteins) back toward expression levels found in animals without nerve injury. The proteomic effects correlate well with the larger and significant relief of pain-like behavior experienced by animals treated with DTMP compared to what they experienced with LR-SCS treatment. From a mechanistic perspective, the multiplexing of pulsed signals provides a more effective way to reduce the neuroinflammatory process mediated by activated glia than what is produced by single signals at a low rate. Although it may be hypothesized that the effects are solely due to the larger electrical charge dosage provided by DTMP, evidence suggests that other factors are at play. For instance, LR-SCS treatment provided a smaller charge and enhanced expression levels of pro-inflammatory genes associated with microglia activation, while DTMP reversed them toward levels in uninjured animals [57,58]. Given that both neurons and glia are responsive to electrical stimulus, it is plausible to multiplex electrical signals that differentially target the various cell populations present in neural tissue affected by nerve injury. An optimization of electrical parameters in the multiplexed signals could ultimately lead to conditions by which the regulation of ECM components may lead to a modulation of glial activation more effectively. Although our design included parallel control groups that accounted for the effects of the lack of the pain model (No-SNI) and lack of treatment (No-SCS), not including an analysis of the effect of treatment on the dorsal quadrant contralateral to injury as a self-control is a limitation of the study.

## 5. Conclusions

Our data strongly suggest that the ECM is affected by the development of a neuropathic pain model and that DTMP, to a much greater degree than LR-SCS, is able to reverse most of the injury-induced changes. This provides valuable insights into the mechanism behind spinal cord stimulation therapies and can help better guide patient care. The ECM provides an unexplored area of neuromodulation targets.

## Data Availability

The data presented in this study is the minimal dataset that supports the central findings of this study. This data is openly available in FigShare (figshare.com) at https://doi.org/10.6084/m9.figshare.22321126.v1. The full dataset of proteins is not publicly available due to intellectual property restrictions placed by the funding institution. Proteomic and phosphoproteomic fold changes obtained from such dataset are available as Supplementary Materials (Tables S1–S7).

## Supplementary Materials

The following supporting information can be downloaded at: https://www.mdpi.com/article/10.3390/biology12040537/s1, Figure S1: Illustration of a general overview of the ECM composition; Figure S2: Illustration of a general overview of extracellular matrix proteins being affected by either DTMP or LR-SCS; Figure S3: Connectivity map of ECM proteins identified from our proteomic dataset. Table S1: Structural ECM proteins—Fold changes and corresponding *p*-values. Table S2: Structural ECM proteins—Fold changes and corresponding *p*-values. Table S3: ECM cell signaling proteins—Fold changes and corresponding *p*-values. Table S4: Structural ECM phosphoproteins—Fold changes. Table S5: Cell adhesion ECM phosphoproteins—Fold changes. Table S6: Cell junction ECM phosphoproteins—Fold changes. Table S7: Cell signaling ECM phosphoproteins—Fold changes.
