# Supplementary material for: Regulation of Expression of Extracellular Matrix Proteins by Differential Target Multiplexed Spinal Cord Stimulation (SCS) and Traditional Low-Rate SCS in a Rat Nerve Injury Model
Source: Biology (Basel). 2023 Mar 31;12(4):537. doi: 10.3390/biology12040537 (PMC10135794; doi:10.3390/biology12040537)
Supplement: Supplementary file 1 [file biology-12-00537-s001.zip › Supplementary Figures.pdf]

Supplementary Figures

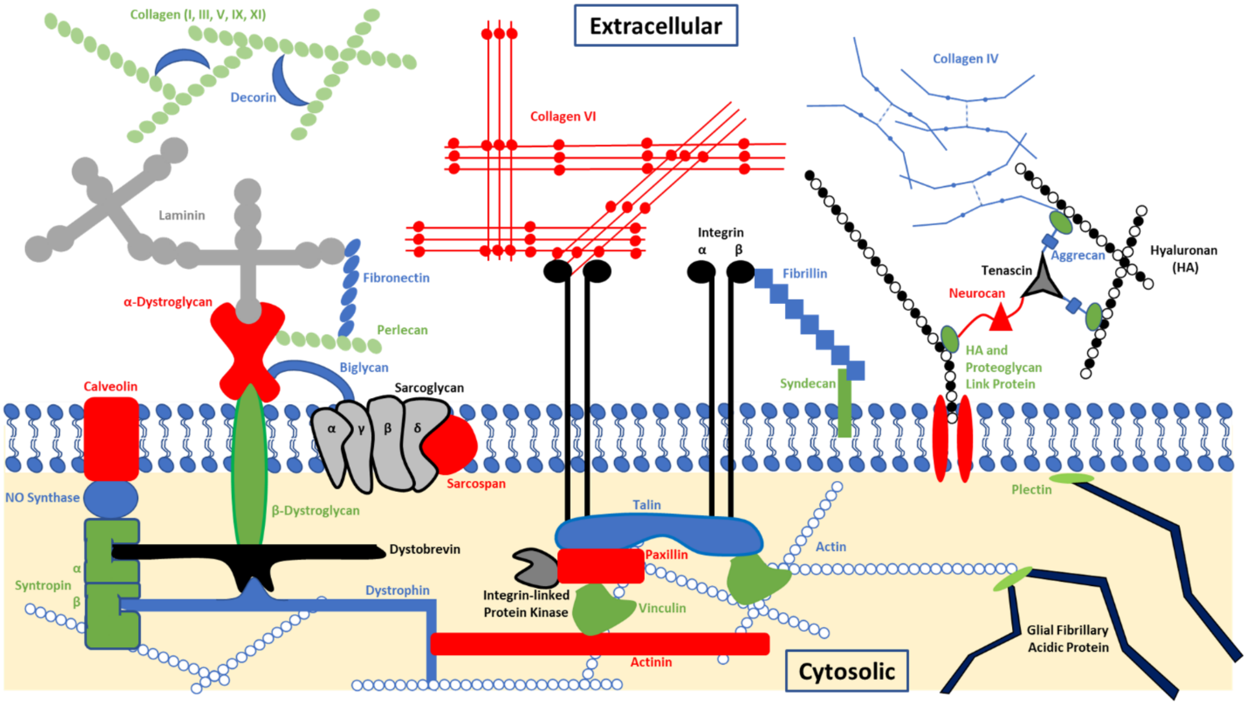

Figure S1. Illustration of a general overview of the ECM composition depicting various extracellular fibers as well as intracellular proteins that help to anchor the ECM. The combination of ECM and ECM-related proteins, such as intracellular networks and signaling proteins, constitute the matrisome. For simplicity, only a few ECM proteins are shown in this illustration.

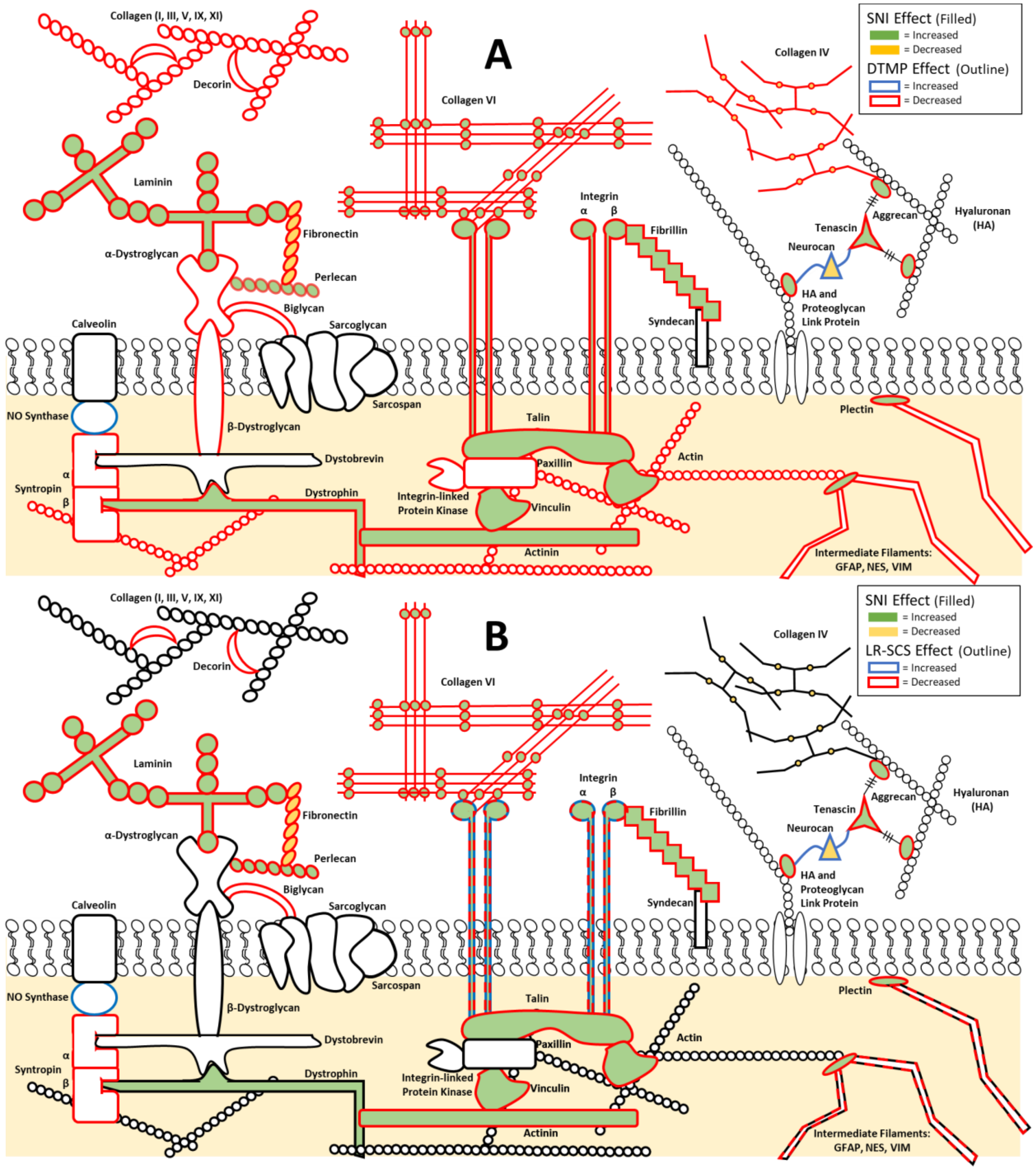

Figure S2. Illustration of a general overview of extracellular matrix proteins being affected by either DTMP **(A)** or LR-SCS **(B)** relative to the effects of the SNI injury model. Black color denotes no effect. In **(B)**, LR-SCS had multiple effects on the four integrin isoforms as well as the intermediate filaments and is thus depicted using dashed colored lines.

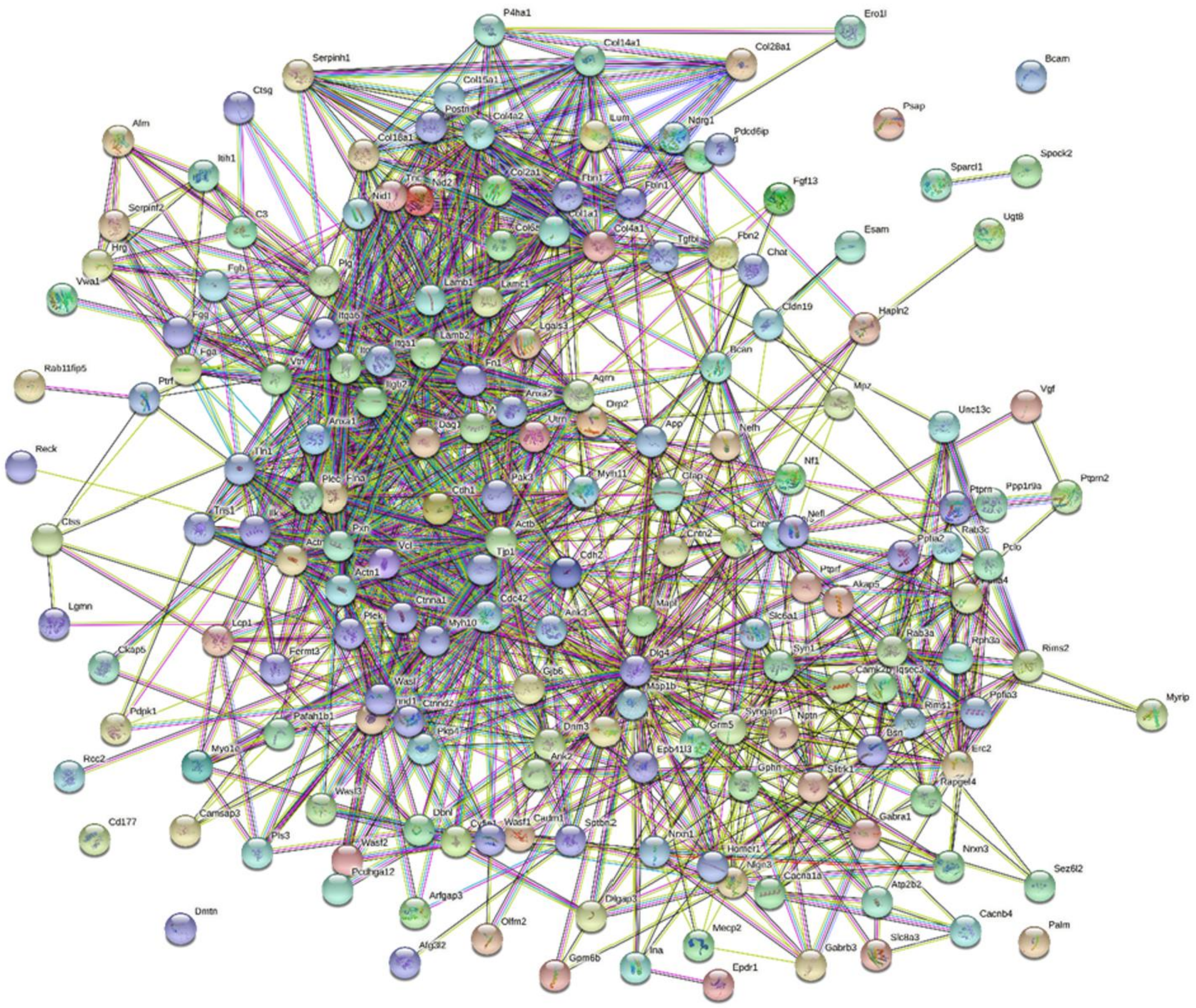

Figure S3. Connectivity map of ECM proteins identified from our proteomic dataset generated using the String database for all 186 proteins. Each line indicates a source of information linking any two proteins in peer-reviewed literature.
