# Supplementary material for: Regulation of Expression of Extracellular Matrix Proteins by Differential Target Multiplexed Spinal Cord Stimulation (SCS) and Traditional Low-Rate SCS in a Rat Nerve Injury Model
Source: Biology (Basel). 2023 Mar 31;12(4):537. doi: 10.3390/biology12040537 (PMC10135794; doi:10.3390/biology12040537)
Supplement: Supplementary file 1 [file biology-12-00537-s001.zip › TableS1.pdf]

Table S1. Structural ECM Proteins - Fold Changes and Corresponding p-Values

Protein Label	Protein Name	No-SCS / No-SNI	p-value	DTMP / No-SCS	p-value	LR-SCS / No-SCS	p-value
CTSS	cathepsin S preproprotein	0.71	0.048	1.65	0.010	2.35	0.008
MYH11	myosin-11	1.26	<0.001	1.27	<0.001	1.09	0.003
GPM6B	neuronal membrane glycoprotein M6-b isoform X5	0.88	0.269	1.23	0.046	1.13	0.220
MYO1E	unconventional myosin-1e	0.93	0.094	1.13	0.014	1.28	0.005
SPOCK2	testican-2 precursor	0.97	0.350	1.10	0.002	1.07	0.210
APP	amyloid-beta A4 protein precursor	0.92	0.016	1.06	0.006	1.08	0.033
ERO1A	ERO1-like protein alpha precursor	0.94	0.118	1.05	0.030	1.02	0.471
NF1	neurofibromin	1.02	0.308	1.02	0.004	1.03	0.059
ACTG1	actin, cytoplasmic 2	1.03	0.406	0.91	0.009	0.95	0.196
Plectin-1 iso 1	plectin isoform 1	1.05	0.001	0.80	<0.001	0.91	<0.001
HAPLN2	hyaluronan and proteoglycan link protein 2 isoform X1	1.11	0.048	0.79	0.001	0.97	0.722
ANXA2	annexin A2	0.93	0.124	0.78	<0.001	1.06	0.154
L-Plastin	plastin-2	1.05	0.434	0.78	0.005	1.03	0.469
DAG1	dystroglycan precursor	1.08	0.356	0.75	0.025	0.92	0.458
FMOD	fibromodulin precursor	1.45	0.021	0.75	0.030	0.53	0.024
COL28A1	collagen alpha-1(XXVIII) chain isoform X1	1.09	0.173	0.75	0.018	0.87	0.105
GFAP	glial fibrillary acidic protein	1.01	0.876	0.74	<0.001	0.97	0.761
RECK	reversion-inducing cysteine-rich protein with Kazal motifs isoform X1	1.17	0.051	0.72	0.006	0.80	0.174
COL4A2	collagen alpha-2(IV) chain isoform X1	0.95	0.358	0.68	0.002	0.95	0.431
LAMB2	laminin subunit beta-2 precursor	1.01	0.745	0.68	<0.001	0.92	0.012
NID1	nidogen-1 isoform X1	1.07	0.221	0.67	<0.001	0.87	0.033
COL1A1	collagen alpha-1(I) chain precursor	0.87	0.062	0.64	<0.001	0.89	0.245
P4HA1	prolyl 4-hydroxylase subunit alpha-1 precursor	1.11	0.100	0.63	0.017	0.76	0.033
COL15A1	collagen alpha-1(XV) chain precursor	1.13	0.008	0.62	<0.001	0.93	0.204
COL4A1	collagen alpha-1(IV) chain precursor	0.70	0.001	0.60	<0.001	1.11	0.172
LAMB1	laminin subunit beta-1 isoform X2	1.01	0.853	0.59	<0.001	0.87	0.029
VWA1	von Willebrand factor A domain-containing protein 1 precursor	1.20	0.027	0.59	0.002	0.82	0.005
LAMC1	laminin subunit gamma-1 precursor	1.07	0.016	0.58	<0.001	0.84	<0.001
FGG	fibrinogen gamma chain precursor	0.45	<0.001	0.57	<0.001	0.78	<0.001
COL14A1	collagen alpha-1(XIV) chain precursor	1.31	<0.001	0.56	<0.001	0.56	<0.001
FGB	fibrinogen beta chain precursor	0.41	<0.001	0.55	<0.001	0.75	<0.001
COL6A1	collagen alpha-1(VI) chain	1.27	0.003	0.55	<0.001	0.66	<0.001
Galectin-3	galectin-3	0.92	0.370	0.53	0.001	1.17	0.101
LUM	lumican precursor	1.10	0.122	0.53	<0.001	0.70	0.002
Nestin iso 1	nestin isoform 1	1.71	<0.001	0.51	<0.001	0.68	<0.001
TGFBI	transforming growth factor-beta-induced protein ig-h3 precursor	1.22	0.118	0.50	<0.001	0.67	0.004
FGA iso 1	fibrinogen alpha chain isoform 1 precursor	0.52	<0.001	0.50	<0.001	0.70	<0.001
SERPINH1	serpin H1 precursor	1.31	<0.001	0.49	<0.001	0.70	0.001
COL18A1	collagen alpha-1(XVIII) chain precursor	1.22	0.023	0.47	0.003	0.73	0.008
ITIH1	inter-alpha-trypsin inhibitor heavy chain H1 precursor	0.95	0.137	0.47	<0.001	0.70	<0.001
PLG	plasminogen precursor	1.06	0.141	0.44	<0.001	0.61	<0.001
SERPINF2	alpha-2-antiplasmin precursor	1.38	0.054	0.44	<0.001	0.57	0.001
VTN	vitronectin precursor	0.90	0.501	0.43	0.003	0.58	0.013
COL2A1	collagen alpha-1(II) chain precursor	0.86	0.186	0.42	0.025	0.94	0.413
FN1	fibronectin precursor	0.78	<0.001	0.37	<0.001	0.67	<0.001
FBLN1	fibulin-1 precursor	1.37	0.018	0.36	0.006	0.72	0.042
CTSG	cathepsin G precursor	0.43	0.010	0.27	0.012	0.14	0.011
POSTN	periostin precursor	1.66	<0.001	0.25	<0.001	0.70	<0.001
