# Supplementary material for: Regulation of Expression of Extracellular Matrix Proteins by Differential Target Multiplexed Spinal Cord Stimulation (SCS) and Traditional Low-Rate SCS in a Rat Nerve Injury Model
Source: Biology (Basel). 2023 Mar 31;12(4):537. doi: 10.3390/biology12040537 (PMC10135794; doi:10.3390/biology12040537)
Supplement: Supplementary file 1 [file biology-12-00537-s001.zip › TableS2.pdf]

Table S2. ECM Cell Junction Proteins - Fold Changes and Corresponding p-Values

Protein Label	Protein Name	No-SCS / No-SNI
CACNB4	voltage-dependent L-type calcium channel subunit beta-4	0.82
SPARCL1	SPARC-like protein 1 precursor	0.90
RAB3A	ras-related protein Rab-3A	0.87
TAU	microtubule-associated protein tau isoform X11	0.87
FGF13	fibroblast growth factor 13	0.86
MGLUR5	metabotropic glutamate receptor 5 precursor	0.91
UGT8	2-hydroxyacylsphingosine 1-beta-galactosyltransferase precursor	0.92
Piccolo iso 1	protein piccolo isoform 1	0.92
GJC3	gap junction gamma-3 protein isoform X1	0.88
HOMER1	homer protein homolog 1	0.90
RIMS1	regulating synaptic membrane exocytosis protein 1	0.86
CAMSAP3	calmodulin-regulated spectrin-associated protein 3 isoform X5	0.89
RIMS2 iso 1	regulating synaptic membrane exocytosis protein 2 isoform 1	0.97
SAPAP3 iso 3	disks large-associated protein 3 isoform 3	0.97
NLGN3	neuroligin-3 precursor	0.89
SEZ6L2	seizure 6-like protein 2 precursor	0.99
Liprin alpha 2	liprin-alpha-2	0.99
PRR14	methyl-CpG-binding protein 2	0.93
NRXN3	neurexin-3 precursor	0.95
GJB6	gap junction beta-6 protein	1.02
Rabphilin 3A	rabphilin-3A isoform X2	0.94
Neuroplastin	neuroplastin precursor	0.89
WASF3	wiskott-Aldrich syndrome protein family member 3 isoform X1	0.93
TAPBP iso 2	ras/Rap GTPase-activating protein SynGAP isoform 2	1.00
WAVE1	wiskott-Aldrich syndrome protein family member 1	0.79
CDC42	cell division control protein 42 homolog precursor	0.85
UNC13C	protein unc-13 homolog C	0.95
CAMK2B iso2	calcium/calmodulin-dependent protein kinase type II subunit beta iso	0.96
Gephyrin	gephyrin	0.92
SLITRK1	SLIT and NTRK-like protein 1 precursor	1.01
CADM1	cell adhesion molecule 1 isoform X6	0.94
N-WASP	neural Wiskott-Aldrich syndrome protein	0.95
PAK3	serine/threonine-protein kinase PAK 3	0.91
BSN	protein bassoon	0.96
SLC8A3	sodium/calcium exchanger 3 precursor	0.94
Neurabin 1	neurabin-1	0.99
AFG3L2	AFG3-like protein 2	0.95
PTPRD	receptor-type tyrosine-protein phosphatase S precursor	0.98
DBNL iso 3	drebrin-like protein isoform 3	0.94
DYN3	dynamin-3 isoform X2	0.98
NCAM-L1 iso2	neural cell adhesion molecule L1 isoform X2	0.95

IQSEC3	IQ motif and SEC7 domain-containing protein 3	0.99
Paralemmin	paralemmin-1	0.89
CYFIP1	cytoplasmic FMR1-interacting protein 1	1.00
GABRB3	gamma-aminobutyric acid receptor subunit beta-3 precursor	0.97
PDK1	3-phosphoinositide-dependent protein kinase 1	0.96
CNTNAP1	contactin-associated protein 1 precursor	0.86
ANK2	LOW QUALITY PROTEIN: ankyrin-2 isoform X1	0.95
CDH2	cadherin-2 precursor	0.96
BCAN iso 1	brevican core protein isoform 1 precursor	0.95
ATP2B2	plasma membrane calcium-transporting ATPase 2	0.91
CACNA1A	voltage-dependent P/Q-type calcium channel subunit alpha-1A	1.00
ERC2	ERC protein 2	0.96
PSD-95	disks large homolog 4	0.97
SYN1	synapsin-1 isoform b	0.98
PPFIA3	liprin-alpha-3	0.89
NRXN1	neurexin-1 precursor	1.01
Liprin alpha 4	liprin-alpha-4	1.01
APP	amyloid-beta A4 protein precursor	0.92
CTNND2	catenin delta-2	0.97
MAP1B	microtubule-associated protein 1B	0.93
Plakophilin 4	plakophilin-4 isoform X9	1.00
ANK3 iso 2	ankyrin-3 isoform 2	0.98
ALIX	programmed cell death 6-interacting protein	0.96
PTPRF	receptor-type tyrosine-protein phosphatase F precursor	1.01
Cntn2	contactin-2 isoform X1	0.97
PLS3	plastin-3 isoform X1	1.00
CKAP5	cytoskeleton-associated protein 5 isoform X1	0.98
EPB41L3	band 4.1-like protein 3 isoform X8	0.97
MYH10	myosin-10 isoform X2	1.03
MYO5A	unconventional myosin-Va	1.02
SPTBN2	spectrin beta chain, non-erythrocytic 2	0.99
PCDHGC3	protocadherin gamma-C3	1.03
GABRA1	gamma-aminobutyric acid receptor subunit alpha-1 precursor	1.04
Claudin-19	claudin-19	0.58
ACTG1	actin, cytoplasmic 2	1.03
ACTN1	alpha-actinin-1	1.09
HAPLN4	hyaluronan and proteoglycan link protein 4 precursor	1.12
ESAM	endothelial cell-selective adhesion molecule precursor	1.16
CTNND1	catenin delta-1	1.03
ZO1	tight junction protein ZO-1 isoform X5	1.09
ACTN4	alpha-actinin-4	1.08
AGRN	agrin precursor	1.22
NFH	neurofilament heavy polypeptide	1.04
Plectin-1 iso 1	plectin isoform 1	1.05
RCC2	protein RCC2	1.17

WAVE2	wiskott-Aldrich syndrome protein family member 2	1.05
NDRG1	protein NDRG1	0.92
Vinculin	vinculin	1.11
Tensin 1	tensin-1	1.17
INA	alpha-internexin	1.20
CHAT	choline O-acetyltransferase	1.19
CTNNA1	catenin alpha-1	1.09
NFL	neurofilament light polypeptide	1.08
PXN	paxillin	1.06
LGMN	legumain precursor	1.18
Talin 1	talin-1	1.06
LAMB2	laminin subunit beta-2 precursor	1.01
CD177	CD177 antigen precursor	0.45
FLNA	filamin-A	1.14
Utrophin	utrophin	1.12
ITGB4	integrin beta-4 precursor	1.05
COL4A1	collagen alpha-1(IV) chain precursor	0.70
LAMC1	laminin subunit gamma-1 precursor	1.07
DRP2	dystrophin-related protein 2 isoform X3	0.95
Myelin P0	myelin protein P0 isoform L-MPZ precursor	0.85
TNC	tenascin precursor	1.35
APOE	apolipoprotein E precursor	1.27
CDH1	cadherin-1 precursor	1.20
NRG1	histidine-rich glycoprotein precursor	1.15
C3	complement C3 precursor	1.40
FN1	fibronectin precursor	0.78

p-value	DTMP / No-SCS	p-value	LR-SCS / No-SCS	p-value
0.090	1.34	0.003	1.30	0.023
0.176	1.32	0.005	0.81	0.047
0.006	1.23	<0.001	1.05	0.372
0.014	1.23	<0.001	1.16	0.057
0.105	1.22	0.033	1.39	0.072
0.011	1.22	0.001	1.15	0.001
0.253	1.21	0.008	1.12	0.138
<0.001	1.21	<0.001	1.12	<0.001
0.199	1.21	0.008	1.22	0.005
0.014	1.21	<0.001	1.15	0.001
0.070	1.20	0.030	1.20	0.037
0.066	1.20	<0.001	1.22	0.002
0.514	1.19	0.015	1.09	0.034
0.665	1.18	0.043	1.06	0.447
0.270	1.18	0.019	1.12	0.131
0.877	1.17	0.045	1.04	0.534
0.723	1.17	0.019	1.07	0.371
0.158	1.16	0.004	1.13	0.067
0.263	1.15	0.004	1.04	0.344
0.572	1.15	0.001	1.02	0.461
0.122	1.15	<0.001	1.02	0.446
0.054	1.15	0.015	1.01	0.753
0.462	1.14	0.001	1.14	0.143
0.983	1.14	0.006	0.98	0.939
0.043	1.14	0.030	1.19	0.013
0.116	1.14	0.022	1.17	0.023
0.173	1.14	0.021	1.00	0.831
0.138	1.14	<0.001	1.01	0.454
0.011	1.13	0.001	1.08	0.047
0.206	1.13	0.034	0.98	0.622
0.202	1.13	0.003	1.14	0.091
0.324	1.12	0.017	1.14	0.014
0.060	1.12	<0.001	1.12	0.008
0.305	1.11	0.001	1.03	0.377
0.335	1.11	0.010	1.01	0.781
0.775	1.11	0.003	1.04	0.236
0.203	1.11	0.001	1.02	0.448
0.482	1.11	<0.001	1.09	0.004
0.193	1.11	0.014	1.10	0.080
0.534	1.11	0.002	1.07	0.100
0.257	1.11	0.013	1.08	0.106

0.778	1.10	0.011	0.98	0.953
0.064	1.10	0.049	1.17	0.038
0.978	1.10	0.003	1.07	0.067
0.219	1.10	0.015	1.14	0.143
0.266	1.10	0.027	1.03	0.407
0.006	1.09	0.007	1.14	0.007
0.021	1.09	<0.001	1.08	0.005
0.382	1.09	0.015	1.05	0.417
0.120	1.08	0.001	1.04	0.247
0.008	1.08	0.002	1.03	0.204
0.900	1.08	0.013	0.78	0.476
0.361	1.08	0.002	1.07	0.023
0.382	1.08	0.007	0.98	0.951
0.685	1.08	0.019	0.93	0.380
0.056	1.07	0.029	1.17	0.004
0.938	1.07	0.041	1.00	0.761
0.927	1.07	0.007	1.01	0.493
0.016	1.06	0.006	1.08	0.033
0.328	1.06	0.020	1.01	0.666
0.005	1.06	0.006	1.01	0.550
0.932	1.06	0.012	1.02	0.313
0.569	1.06	0.004	1.06	0.092
0.192	1.05	0.037	1.05	0.140
0.636	1.05	0.038	1.08	0.027
0.173	1.05	0.002	1.00	0.582
0.980	1.05	0.025	0.99	0.972
0.388	1.04	0.035	1.06	0.024
0.313	1.04	0.048	1.03	0.384
0.245	1.02	0.043	0.97	0.597
0.320	1.02	0.004	0.98	0.712
0.523	1.02	0.019	0.98	0.711
0.707	1.02	0.039	1.04	0.245
0.850	0.95	0.046	1.07	0.154
0.018	0.94	0.015	1.92	0.072
0.406	0.91	0.009	0.95	0.196
0.033	0.89	0.003	0.99	0.920
0.013	0.89	0.002	0.88	<0.001
0.433	0.88	0.002	0.95	0.756
0.566	0.87	0.041	1.00	0.815
<0.001	0.85	<0.001	0.95	0.132
0.007	0.84	<0.001	0.89	0.002
0.002	0.84	0.024	0.81	0.003
0.433	0.80	<0.001	0.93	0.283
0.001	0.80	<0.001	0.91	<0.001
0.063	0.80	0.029	0.86	0.065

0.484	0.80	0.009	1.01	0.722
0.309	0.78	0.001	1.02	0.539
<0.001	0.78	<0.001	0.84	<0.001
<0.001	0.78	<0.001	0.89	0.019
<0.001	0.78	<0.001	0.75	<0.001
0.132	0.77	0.046	0.60	0.031
0.091	0.77	0.001	0.94	0.409
0.220	0.76	<0.001	0.85	0.002
0.347	0.74	0.007	0.97	0.865
0.185	0.71	0.031	0.88	0.071
0.002	0.69	<0.001	0.89	<0.001
0.745	0.68	<0.001	0.92	0.012
0.034	0.62	0.007	0.23	0.048
<0.001	0.62	<0.001	0.82	<0.001
<0.001	0.61	<0.001	0.83	<0.001
0.107	0.60	<0.001	0.89	0.004
0.001	0.60	<0.001	1.11	0.172
0.016	0.58	<0.001	0.84	<0.001
0.243	0.57	<0.001	1.06	0.127
<0.001	0.57	<0.001	0.92	0.037
<0.001	0.56	<0.001	0.55	<0.001
<0.001	0.53	<0.001	0.58	<0.001
0.090	0.50	0.047	0.81	0.103
0.020	0.46	<0.001	0.60	<0.001
<0.001	0.39	<0.001	0.57	<0.001
<0.001	0.37	<0.001	0.67	<0.001
