# Supplementary material for: Regulation of Expression of Extracellular Matrix Proteins by Differential Target Multiplexed Spinal Cord Stimulation (SCS) and Traditional Low-Rate SCS in a Rat Nerve Injury Model
Source: Biology (Basel). 2023 Mar 31;12(4):537. doi: 10.3390/biology12040537 (PMC10135794; doi:10.3390/biology12040537)
Supplement: Supplementary file 1 [file biology-12-00537-s001.zip › TableS3.pdf]

Table S3. ECM Cell Signaling Proteins - Fold Changes and Corresponding p-Values

Protein Label	Protein Name	No-SCS / No-SNI	p-value	DTMP / No-SCS	p-value	LR-SCS / No-SCS	p-value
CACNB4	voltage-dependent L-type calcium channel subunit beta-4	0.82	0.090	1.34	0.003	1.30	0.023
CTSS	cathepsin S preproprotein	0.71	0.048	1.32	0.023	2.35	0.008
SPARCL1	SPARC-like protein 1 precursor	0.90	0.176	1.32	0.005	0.81	0.047
PSAP	prosaposin isoform D preproprotein	0.82	0.002	1.28	0.001	1.42	0.001
RAB3A	ras-related protein Rab-3A	0.87	0.006	1.23	<0.001	1.05	0.372
TAU	microtubule-associated protein tau isoform X11	0.87	0.014	1.23	<0.001	1.16	0.057
FGF13	fibroblast growth factor 13	0.86	0.105	1.22	0.033	1.39	0.072
MGLUR5	metabotropic glutamate receptor 5 precursor	0.91	0.011	1.22	0.001	1.15	0.001
DMTN	dematin isoform X1	0.84	0.010	1.22	0.011	1.43	0.020
PTPRN2	receptor-type tyrosine-protein phosphatase N2 precursor	0.99	0.606	1.21	<0.001	1.06	0.129
Piccolo iso 1	protein piccolo isoform 1	0.92	<0.001	1.21	<0.001	1.12	<0.001
GJC3	gap junction gamma-3 protein isoform X1	0.88	0.199	1.21	0.008	1.22	0.005
HOMER1	homer protein homolog 1	0.90	0.014	1.21	<0.001	1.15	0.001
RIMS1	regulating synaptic membrane exocytosis protein 1	0.86	0.070	1.20	0.030	1.20	0.037
RIMS2 iso 1	regulating synaptic membrane exocytosis protein 2 isoform 1	0.97	0.514	1.19	0.015	1.09	0.034
SAPAP3 iso 3	disks large-associated protein 3 isoform 3	0.97	0.665	1.18	0.043	1.06	0.447
NLGN3	neuroligin-3 precursor	0.89	0.270	1.18	0.019	1.12	0.131
PTPRN	receptor-type tyrosine-protein phosphatase-like N precursor	0.97	0.590	1.17	0.041	1.10	0.199
VGF	neurosecretory protein VGF precursor	0.99	0.603	1.16	0.009	1.18	0.021
AKAP5	A-kinase anchor protein 5	0.88	0.061	1.16	0.046	1.17	0.245
PRR14	methyl-CpG-binding protein 2	0.93	0.158	1.16	0.004	1.13	0.067
GJB6	gap junction beta-6 protein	1.02	0.572	1.15	0.001	1.02	0.461
Rabphilin 3A	rabphilin-3A isoform X2	0.94	0.122	1.15	<0.001	1.02	0.446
Neuroplastin	neuroplastin precursor	0.89	0.054	1.15	0.015	1.01	0.753
RAB11FIP5	rab11 family-interacting protein 5 isoform X1	0.94	0.162	1.15	0.001	1.18	0.028
TAPBP iso 2	ras/Rap GTPase-activating protein SynGAP isoform 2	1.00	0.983	1.14	0.006	0.98	0.939
WAVE1	wiskott-Aldrich syndrome protein family member 1	0.79	0.043	1.14	0.030	1.19	0.013
CDC42	cell division control protein 42 homolog precursor	0.85	0.116	1.14	0.022	1.17	0.023
UNC13C	protein unc-13 homolog C	0.95	0.173	1.14	0.021	1.00	0.831
CAMK2B iso 2	calcium/calmodulin-dependent protein kinase type II subunit beta iso2	0.96	0.138	1.14	<0.001	1.01	0.454
MYO1E	unconventional myosin-Ie	0.93	0.094	1.13	0.014	1.28	0.005
CADM1	cell adhesion molecule 1 isoform X6	0.94	0.202	1.13	0.003	1.14	0.091
PAK3	serine/threonine-protein kinase PAK 3	0.91	0.060	1.12	<0.001	1.12	0.008
BSN	protein bassoon	0.96	0.305	1.11	0.001	1.03	0.377
Neurabin 1	neurabin-1	0.99	0.775	1.11	0.003	1.04	0.236
PTPRD	receptor-type tyrosine-protein phosphatase S precursor	0.98	0.482	1.11	<0.001	1.09	0.004
DBNL iso 3	drebrin-like protein isoform 3	0.94	0.193	1.11	0.014	1.10	0.080
NCAM-L1 iso2	neural cell adhesion molecule L1 isoform X2	0.95	0.257	1.11	0.013	1.08	0.106
Paralemmin	paralemmin-1	0.89	0.064	1.10	0.049	1.17	0.038
CYFIP1	cytoplasmic FMR1-interacting protein 1	1.00	0.978	1.10	0.003	1.07	0.067
GABRB3	gamma-aminobutyric acid receptor subunit beta-3 precursor	0.97	0.219	1.10	0.015	1.14	0.143
RAPGEF4	rap guanine nucleotide exchange factor 4 isoform X1	1.00	0.981	1.10	0.004	1.03	0.179
OLFM2	noelin-2 precursor	0.98	0.536	1.10	0.047	0.97	0.714
PDK1	3-phosphoinositide-dependent protein kinase 1	0.96	0.266	1.10	0.027	1.03	0.407
CNTNAP1	contactin-associated protein 1 precursor	0.86	0.006	1.09	0.007	1.14	0.007
ANK2	ankyrin-2 isoform X1	0.95	0.021	1.09	<0.001	1.08	0.005
CACNA1A	voltage-dependent P/Q-type calcium channel subunit alpha-1A	1.00	0.900	1.08	0.013	0.78	0.476
ERC2	ERC protein 2	0.96	0.361	1.08	0.002	1.07	0.023
PSD-95	disks large homolog 4	0.97	0.382	1.08	0.007	0.98	0.951
SYN1	synapsin-1 isoform b	0.98	0.685	1.08	0.019	0.93	0.380
PPFIA3	liprin-alpha-3	0.89	0.056	1.07	0.029	1.17	0.004
NRXN1	neurexin-1 precursor	1.01	0.938	1.07	0.041	1.00	0.761
APP	amyloid-beta A4 protein precursor	0.92	0.016	1.06	0.006	1.08	0.033
ANK3 iso 2	ankyrin-3 isoform 2	0.98	0.569	1.06	0.004	1.06	0.092
PTPRF	receptor-type tyrosine-protein phosphatase F precursor	1.01	0.636	1.05	0.038	1.08	0.027

CNTN2	contactin-2 isoform X1	0.97	0.173	1.05	0.002	1.00	0.582
ERO1A	ERO1-like protein alpha precursor	0.94	0.118	1.05	0.030	1.02	0.471
CKAP5	cytoskeleton-associated protein 5 isoform X1	0.98	0.388	1.04	0.035	1.06	0.024
PAFAH1B1	platelet-activating factor acetylhydrolase IB subunit alpha	0.94	0.121	1.03	0.019	1.01	0.553
MYO5A	unconventional myosin-Va	1.02	0.320	1.02	0.004	0.98	0.712
SPTBN2	spectrin beta chain, non-erythrocytic 2	0.99	0.523	1.02	0.019	0.98	0.711
NF1	neurofibromin	1.02	0.308	1.02	0.004	1.03	0.059
GABRA1	gamma-aminobutyric acid receptor subunit alpha-1 precursor	1.04	0.850	0.95	0.046	1.07	0.154
Claudin-19	claudin-19	0.58	0.018	0.94	0.015	1.92	0.072
HAPLN4	hyaluronan and proteoglycan link protein 4 precursor	1.12	0.013	0.89	0.002	0.88	<0.001
ACTN4	alpha-actinin-4	1.08	0.007	0.84	<0.001	0.89	0.002
AGRN	agrin precursor	1.22	0.002	0.84	0.024	0.81	0.003
ILK	integrin-linked protein kinase	1.04	0.430	0.81	0.001	0.94	0.256
Plectin-1 iso 1	plectin isoform 1	1.05	0.001	0.80	<0.001	0.91	<0.001
RCC2	protein RCC2	1.17	0.063	0.80	0.029	0.86	0.065
WAVE2	wiskott-Aldrich syndrome protein family member 2	1.05	0.484	0.80	0.009	1.01	0.722
NDRG1	protein NDRG1	0.92	0.309	0.78	0.001	1.02	0.539
L-plastin	plastin-2	1.05	0.434	0.78	0.005	1.03	0.469
CHAT	choline O-acetyltransferase	1.19	0.132	0.77	0.046	0.60	0.031
CTNNA1	catenin alpha-1	1.09	0.091	0.77	0.001	0.94	0.409
DAG1	dystroglycan precursor	1.08	0.356	0.75	0.025	0.92	0.458
ITGB2	integrin beta-2 precursor	0.94	0.266	0.75	0.002	1.22	0.005
PXN	paxillin	1.06	0.347	0.74	0.007	0.97	0.865
RECK	reversion-inducing cysteine-rich protein with Kazal motifs isoform X1	1.17	0.051	0.72	0.006	0.80	0.174
ITGA1	integrin alpha-1 precursor	1.39	0.004	0.70	0.006	0.74	0.031
ITGA6	integrin alpha-6 precursor	1.09	0.083	0.69	<0.001	0.91	0.060
Talin 1	talin-1	1.06	0.002	0.69	<0.001	0.89	<0.001
COL4A2	collagen alpha-2(IV) chain isoform X1	0.95	0.358	0.68	0.002	0.95	0.431
Kindlin-3	fermitin family homolog 3	0.94	0.425	0.66	0.003	0.96	0.756
COL1A1	collagen alpha-1(I) chain precursor	0.87	0.062	0.64	<0.001	0.89	0.245
Pleckstrin	pleckstrin	0.70	0.007	0.64	0.014	1.11	0.253
FLNA	filamin-A	1.14	<0.001	0.62	<0.001	0.82	<0.001
ITGB4	integrin beta-4 precursor	1.05	0.107	0.60	<0.001	0.89	0.004
COL4A1	collagen alpha-1(IV) chain precursor	0.70	0.001	0.60	<0.001	1.11	0.172
DRP2	dystrophin-related protein 2 isoform X3	0.95	0.243	0.57	<0.001	1.06	0.127
TNC	tenascin precursor	1.35	<0.001	0.56	<0.001	0.55	<0.001
APOE	apolipoprotein E precursor	1.27	<0.001	0.53	<0.001	0.58	<0.001
FBN2	fibrillin-2 precursor	1.15	0.159	0.51	0.022	0.84	0.181
CDH1	cadherin-1 precursor	1.20	0.090	0.50	0.047	0.81	0.103
NRG1	histidine-rich glycoprotein precursor	1.15	0.020	0.46	<0.001	0.60	<0.001
FBN1	fibrillin-1 isoform X1	1.26	<0.001	0.45	<0.001	0.82	<0.001
PLG	plasminogen precursor	1.06	0.141	0.44	<0.001	0.61	<0.001
COL2A1	collagen alpha-1(II) chain precursor	0.86	0.186	0.42	0.025	0.94	0.413
C3	complement C3 precursor	1.40	<0.001	0.39	<0.001	0.57	<0.001
ANXA1	annexin A1	0.96	0.474	0.38	<0.001	0.52	<0.001
FN1	fibronectin precursor	0.78	<0.001	0.37	<0.001	0.67	<0.001
CTSG	cathepsin G precursor	0.43	0.010	0.27	0.012	0.14	0.011
