# Supplementary material for: Regulation of Expression of Extracellular Matrix Proteins by Differential Target Multiplexed Spinal Cord Stimulation (SCS) and Traditional Low-Rate SCS in a Rat Nerve Injury Model
Source: Biology (Basel). 2023 Mar 31;12(4):537. doi: 10.3390/biology12040537 (PMC10135794; doi:10.3390/biology12040537)
Supplement: Supplementary file 1 [file biology-12-00537-s001.zip › TableS4.pdf]

Table S4. Structural ECM Phosphoproteins - Fold Changes

Protein	Phosphoprotein Isoform	No-SCS / No-SNI	DTMP / No-SCS	LR-SCS / No-SCS
PLECTIN	p-PLECTIN-1 (UNK)	0.63	2.12	2.67
	p-PLECTIN-1 (UNK)	0.80	0.87	1.12
	p-PLECTIN-1 (UNK)	0.89	1.10	1.19
	p-PLECTIN-1 (UNK)	0.90	0.13	0.53
	p-PLECTIN-1 (UNK)	1.05	0.85	1.03
	p-PLECTIN-1 (UNK)	1.14	0.83	1.22
	p-PLECTIN-1 (UNK)	1.17	0.63	0.53
	p-PLECTIN-1 (UNK)	1.20	0.55	0.68
	p-PLECTIN-1 (UNK)	1.21	0.88	1.07
	p-PLECTIN-1 (UNK)	1.25	0.82	1.53
	p-PLECTIN-1 (UNK)	1.52	0.42	0.33
	p-PLECTIN-1 (UNK)	2.90	1.04	4.70
	p-PLECTIN-1 (21)	3.66	0.45	1.83
	p-PLECTIN-1 (UNK)	4.12	0.84	2.27
	p-PLECTIN-1 (UNK)	5.12	0.61	1.42
	p-PLECTIN-1 (UNK)	6.06	0.24	0.88
	p-PLECTIN-1 (UNK)	13.61	0.24	1.27
GFAP	p-GFAP (13)	0.83	2.47	4.17
	p-GFAP (36, 41)	0.89	1.52	1.67
	p-GFAP (267)	0.91	0.94	1.77
	p-GFAP (15)	1.01	1.36	2.93
	p-GFAP (383)	1.02	0.91	1.34
	p-GFAP (41)	1.04	1.33	1.84
	p-GFAP (38)	1.20	1.25	1.46
	p-GFAP (321)	1.30	0.84	1.15
	p-GFAP (296)	1.43	1.44	2.17
	p-GFAP (303)	1.51	0.23	0.34
	p-GFAP (398)	1.61	0.85	1.58
	p-GFAP (12)	1.66	1.35	2.51
	p-GFAP (391)	1.78	0.98	1.74
	p-GFAP (319)	2.11	0.69	0.98
	p-GFAP (36)	2.15	0.76	0.97
	p-GFAP (31)	2.46	0.68	2.21
	p-GFAP (148)	6.40	0.64	0.58
NESTIN	NESTIN (729)	8.32	0.15	0.56
	NESTIN (620)	7.70	0.19	0.42
	NESTIN (773)	4.98	0.19	0.84
	NESTIN (1016)	3.76	0.54	0.60
	NESTIN (1866)	3.38	0.28	0.71
	NESTIN (924)	3.16	0.44	1.19
	NESTIN (1166)	2.37	0.31	0.67
	NESTIN (316)	2.24	1.07	0.91
	NESTIN (630)	2.23	0.33	0.68
	NESTIN (1594)	2.18	0.34	0.91
	NESTIN (1606)	1.97	0.20	0.99
	NESTIN (861)	1.55	0.71	1.86
	NESTIN (723, 729)	1.21	1.04	1.12
	NESTIN (723, 726)	0.92	1.77	0.93
NF1	p-NF1 (866)	0.83	1.25	1.07
	p-NF1 (2496)	0.89	0.90	1.14
	p-NF1 (2783)	0.97	1.15	0.42
	p-NF1 (2524)	1.00	1.05	1.22
	p-NF1 (2495)	1.07	0.90	0.84
	p-NF1 (2580)	1.20	0.57	0.82
	p-NF1 (2578)	1.21	0.56	1.08
	p-NF1 (2169)	1.85	0.77	0.49
ACTG1	p-ACTG1 (240;241;242)	0.61	1.21	1.34
	p-ACTG1 (60;61;62)	0.82	0.81	1.20
	p-ACTG1 (203)	1.65	0.87	0.77
	p-ACTG1 (UNK)	1.89	0.81	0.95
	p-ACTG1 (199)	3.98	0.34	0.44
FGA	p-FGA (502;505)	7.15	0.28	0.83
	p-FGA (428;431)	14.10	0.24	0.38
DAG1	p-DAG1 (788)	8.36	0.30	0.20
COL2A1	p-COL2A1 (594;662)	1.94	0.18	0.16
MYO1E	p-MYO1E (1001)	0.86	2.06	0.28
ANXA2	p-ANXA2 (184)	1.09	0.88	1.49
MYH11	p-MYH11 (1954)	0.98	1.10	0.89
SPOCK2	p-SPOCK2 (74;72)	0.91	1.18	1.11

Numbers in parenthesis indicate phosphorylated residues. Comma separation indicates multiple phosphorylation. Semicolon separation indicates possible residue location
