# Supplementary material for: Regulation of Expression of Extracellular Matrix Proteins by Differential Target Multiplexed Spinal Cord Stimulation (SCS) and Traditional Low-Rate SCS in a Rat Nerve Injury Model
Source: Biology (Basel). 2023 Mar 31;12(4):537. doi: 10.3390/biology12040537 (PMC10135794; doi:10.3390/biology12040537)
Supplement: Supplementary file 1 [file biology-12-00537-s001.zip › TableS5.pdf]

Table S5. Cell Adhesion ECM Phosphoproteins - Fold Changes

Protein	Phosphoprotein Isoform	No-SCS / No-SNI	DTMP / No-SCS	LR-SCS / No-SCS
PLAKOPHILIN	p-PLAKOPHILIN 4 (UNK)	0.16	1.47	1.13
	p-PLAKOPHILIN 4 (UNK)	0.25	1.31	3.93
	p-PLAKOPHILIN 4 (UNK)	0.31	1.93	1.38
	p-PLAKOPHILIN 4 (UNK)	0.35	2.56	2.09
	p-PLAKOPHILIN 4 (UNK)	0.37	2.10	2.23
	p-PLAKOPHILIN 4 (UNK)	0.39	2.15	2.69
	p-PLAKOPHILIN 4 (UNK)	0.41	1.73	0.71
	p-PLAKOPHILIN 4 (UNK)	0.56	1.96	2.46
	p-PLAKOPHILIN 4 (UNK)	0.56	0.42	0.61
	p-PLAKOPHILIN 4 (UNK)	0.58	0.81	0.45
	p-PLAKOPHILIN 4 (UNK)	0.59	2.06	1.94
	p-PLAKOPHILIN 4 (UNK)	0.68	1.76	0.39
	p-PLAKOPHILIN 4 (UNK)	0.71	1.10	1.42
	p-PLAKOPHILIN 4 (UNK)	0.71	1.42	2.24
	p-PLAKOPHILIN 4 (UNK)	0.79	1.49	1.48
	p-PLAKOPHILIN 4 (UNK)	0.79	0.47	0.47
	p-PLAKOPHILIN 4 (UNK)	0.81	1.18	1.62
	p-PLAKOPHILIN 4 (143)	0.82	1.06	1.31
	p-PLAKOPHILIN 4 (UNK)	0.87	1.11	0.90
	p-PLAKOPHILIN 4 (UNK)	0.91	0.94	5.40
	p-PLAKOPHILIN 4 (UNK)	0.92	1.43	0.34
	p-PLAKOPHILIN 4 (UNK)	0.94	1.25	1.74
	p-PLAKOPHILIN 4 (UNK)	0.98	1.32	1.04
	p-PLAKOPHILIN 4 (UNK)	1.03	1.36	1.01
	p-PLAKOPHILIN 4 (UNK)	1.04	0.92	0.50
	p-PLAKOPHILIN 4 (UNK)	1.08	0.79	1.44
	p-PLAKOPHILIN 4 (UNK)	1.19	1.22	0.65
ZO1	p-ZO1 (UNK)	0.56	1.45	1.61
	p-ZO1 (UNK)	0.73	1.67	1.23
	p-ZO1 (UNK)	0.92	0.52	1.62
	p-ZO1 (UNK)	1.00	1.10	0.70
	p-ZO1 (UNK)	1.10	1.25	0.58
	p-ZO1 (UNK)	1.12	0.59	0.52
	p-ZO1 (UNK)	1.15	0.46	0.37
	p-ZO1 (UNK)	1.43	0.90	0.67
	p-ZO1 (UNK)	1.75	0.69	0.85
	p-ZO1 (UNK)	1.82	0.74	4.76
	p-ZO1 (UNK)	2.18	0.58	0.79
	p-ZO1 (UNK)	4.42	0.57	0.92
CAMSAP3	p-CAMSAP3 (UNK)	0.24	2.68	3.38
	p-CAMSAP3 (384)	0.37	1.21	0.95
	p-CAMSAP3 (UNK)	0.42	1.63	1.27
	p-CAMSAP3 (UNK)	0.43	1.71	0.27
	p-CAMSAP3 (UNK)	0.55	1.02	2.87
	p-CAMSAP3 (UNK)	0.68	1.28	0.94
	p-CAMSAP3 (UNK)	0.68	1.20	1.35
	p-CAMSAP3 (UNK)	0.76	1.29	1.03

	p-CAMSAP3 (363)	0.78	1.22	0.68
	p-CAMSAP3 (UNK)	1.10	0.90	1.23
	p-CAMSAP3 (UNK)	1.10	0.52	0.58
CTNND2	p-CTNND2 (UNK)	0.25	2.25	1.62
	p-CTNND2 (UNK)	0.30	2.47	2.33
	p-CTNND2 (398;412)	0.41	2.04	3.06
	p-CTNND2 (UNK)	0.47	1.31	1.35
	p-CTNND2 (UNK)	0.73	1.20	1.38
	p-CTNND2 (UNK)	0.84	1.17	0.94
	p-CTNND2 (UNK)	1.02	0.67	0.65
	p-CTNND2 (32)	1.18	0.67	2.36
	p-CTNND2 (UNK)	2.07	0.83	0.49
	p-CTNND2 (UNK)	3.74	1.34	1.92
MYELIN P0	p-MYELIN P0 (226)	0.59	0.56	0.14
	p-MYELIN P0 (195)	0.81	0.99	1.55
	p-MYELIN P0 (216)	0.81	0.85	0.67
	p-MYELIN P0 (294)	1.32	0.71	1.10
	p-MYELIN P0 (292)	1.60	0.45	0.87
	p-MYELIN P0 (294, 297)	6.52	0.65	1.47
	p-MYELIN P0 (78)	7.01	0.31	0.46
	p-MYELIN P0 (106)	11.19	0.21	0.43
CTNND1	p-CTNND1 (UNK)	0.36	3.58	4.18
	p-CTNND1 (349)	0.75	1.07	1.12
	p-CTNND1 (47)	1.06	0.64	0.14
	p-CTNND1 (288)	1.33	0.56	1.11
	p-CTNND1 (252)	1.58	1.16	2.71
	p-CTNND1 (228)	1.89	0.60	0.78
	p-CTNND1 (352)	1.98	0.77	1.44
	p-CTNND1 (268)	17.92	0.40	1.24
SPARCL1	p-SPARCL1 (340)	0.49	3.03	4.68
	p-SPARCL1 (68)	0.74	1.19	0.87
	p-SPARCL1 (68, 76)	0.81	1.32	1.10
	p-SPARCL1 (76)	0.82	1.15	0.80
	p-SPARCL1 (333, 340)	0.95	0.99	0.67
	p-SPARCL1 (68, 77)	1.02	1.23	0.96
	p-SPARCL1 (151)	1.22	1.04	0.30
NCAM-L1	p-NCAM-L1 iso2 (1173, 1180)	0.91	0.37	1.70
	p-NCAM-L1 iso2 (1175;1180)	1.02	1.20	0.63
	p-NCAM-L1 iso2 (1173;1178)	1.06	1.01	0.58
	p-NCAM-L1 iso2 (1178;1183)	1.07	0.92	1.60
	p-NCAM-L1 iso2 (1160, 1174)	1.10	0.85	1.14
	p-NCAM-L1 iso2 (UNK)	1.21	0.93	0.77
	p-NCAM-L1 iso2 (1173, 1183)	1.66	0.86	0.85
ACTG1	p-ACTG1 (240;241;242)	0.61	1.21	1.34
	p-ACTG1 (60;61;62)	0.82	0.81	1.20
	p-ACTG1 (203)	1.65	0.87	0.77
	p-ACTG1 (UNK)	1.89	0.81	0.95
	p-ACTG1 (199)	3.98	0.34	0.44
FLNA	p-FLNA (2172;2180)	0.90	1.07	1.57
	p-FLNA (UNK)	1.08	0.69	1.35
	p-FLNA (968)	1.65	0.71	0.91

	p-FLNA (1459)	2.30	0.34	0.29
	p-FLNA (2144;2152)	2.40	0.50	0.69
VINCULIN	p-VINCULIN (721)	1.54	0.63	0.71
	p-VINCULIN (346)	1.61	0.78	0.84
FGA	p-FGA (502;505)	7.15	0.28	0.83
	p-FGA (428;431)	14.10	0.24	0.38
TALIN 1	p-TALIN 1 (1260;1277)	1.47	0.52	0.67
	p-TALIN 1 (425)	2.24	0.74	1.07
PSD-95	p-PSD-95 (UNK)	0.77	1.16	1.44
	p-PSD-95 (415)	1.31	1.03	0.60
ESAM	p-ESAM (370)	1.26	0.81	0.61
CTNNA1	p-CTNNA1 (643)	1.18	0.70	0.90
CADM1	p-CADM1 (468)	1.32	0.77	0.93

Numbers in parenthesis indicate phosphorylated residues. Comma separation indicates multiple phosphorylation. Semicolon separation indicates possible residue location
