# Supplementary material for: Regulation of Expression of Extracellular Matrix Proteins by Differential Target Multiplexed Spinal Cord Stimulation (SCS) and Traditional Low-Rate SCS in a Rat Nerve Injury Model
Source: Biology (Basel). 2023 Mar 31;12(4):537. doi: 10.3390/biology12040537 (PMC10135794; doi:10.3390/biology12040537)
Supplement: Supplementary file 1 [file biology-12-00537-s001.zip › TableS6.pdf]

Table S6. Cell Junction ECM Phosphoproteins - Fold Changes

Protein	Phosphoprotein Isoform	No-SCS / No-SNI	DTMP / No-SCS	LR-SCS / No-SCS
	p-NFH (1048)	0.32	2.50	4.34
	p-NFH (682)	0.40	1.61	2.64
	p-NFH (622)	0.46	1.64	2.98
	p-NFH (586)	0.49	1.10	2.08
	p-NFH (1051)	0.51	2.25	6.06
	p-NFH (658)	0.55	1.44	2.22
	p-NFH (580)	0.58	1.17	0.71
	p-NFH (838)	0.60	1.21	1.86
	p-NFH (814, 819)	0.62	2.40	7.67
	p-NFH (643, 646)	0.70	5.67	42.65
	p-NFH (1030)	0.75	1.08	1.57
	p-NFH (819)	0.81	1.06	1.67
	p-NFH (712, 724, 730)	0.85	1.20	1.86
	p-NFH (706)	0.86	0.96	1.73
	p-NFH (1034)	0.87	1.98	6.68
	p-NFH (562)	0.87	1.00	1.23
	p-NFH (646)	0.90	1.05	1.28
	p-NFH (43)	0.91	1.16	1.53
	p-NFH (706, 712)	0.93	1.22	2.64
	p-NFH (658, 664, 670)	0.95	1.20	1.37
	p-NFH (61)	0.96	0.96	1.37
	p-NFH (872)	0.99	1.00	1.13
	p-NFH (604)	1.03	0.98	1.27
	p-NFH (562, 568)	1.05	1.45	1.36
	p-NFH (929)	1.07	0.67	0.89
	p-NFH (550, 556)	1.08	1.16	1.19
	p-NFH (724, 730, 736)	1.08	0.64	1.88
	p-NFH (844)	1.10	0.96	1.28
	p-NFH (556)	1.12	0.88	0.94
	p-NFH (694, 700)	1.12	0.85	1.36
	p-NFH (643, 646, 652)	1.13	2.06	0.65
	p-NFH (301)	1.16	0.92	1.04
	p-NFH (838, 844)	1.16	0.82	1.19
	p-NFH (658, 661, 670)	1.17	0.80	1.34
	p-NFH (646, 652, 661)	1.20	2.95	22.88
	p-NFH (794)	1.20	0.83	1.12
	p-NFH (664)	1.22	0.77	1.11
	p-NFH (682, 688, 694)	1.26	0.84	1.66
	p-NFH (700, 706)	1.26	0.77	1.47
	p-NFH (894)	1.27	1.13	1.30
	p-NFH (664, 670, 676)	1.29	0.68	0.87
	p-NFH (124)	1.31	0.33	0.25
	p-NFH (754)	1.32	0.91	1.36
	p-NFH (819, 824)	1.34	0.72	1.05
	p-NFH (844, 852)	1.34	0.76	1.09
	p-NFH (688, 694, 700)	1.36	1.11	1.34
	p-NFH (682, 688)	1.39	0.76	1.25

NFH	p-NFH (748, 754)	1.39	0.83	1.44
	p-NFH (891)	1.39	1.00	1.01
	p-NFH (652)	1.42	4.73	0.87
	p-NFH (646, 652, 658)	1.45	0.74	0.64
	p-NFH (742, 748, 754)	1.45	0.88	1.56
	p-NFH (586, 592, 598)	1.46	0.77	1.09
	p-NFH (580, 586)	1.47	0.79	1.05
	p-NFH (76)	1.47	0.44	0.46
	p-NFH (538, 544)	1.48	0.72	0.89
	p-NFH (74)	1.49	0.45	0.46
	p-NFH (742, 748)	1.49	0.70	1.21
	p-NFH (54)	1.50	0.97	3.17
	p-NFH (643, 652)	1.51	0.74	2.44
	p-NFH (604, 610)	1.51	0.83	1.13
	p-NFH (574, 583)	1.52	1.28	6.63
	p-NFH (63)	1.54	1.01	1.27
	p-NFH (684, 688, 694)	1.55	0.80	1.33
	p-NFH (640, 646)	1.55	0.74	1.06
	p-NFH (510, 520, 526)	1.55	0.67	0.86
	p-NFH (670, 676)	1.57	0.67	0.91
	p-NFH (526, 532, 538)	1.58	0.74	1.01
	p-NFH (670, 676, 682)	1.60	0.74	1.09
	p-NFH (774)	1.60	0.83	1.28
	p-NFH (574, 580)	1.61	0.64	0.72
	p-NFH (658, 664)	1.64	0.73	1.12
	p-NFH (520, 526, 532)	1.64	0.63	0.78
	p-NFH (694, 700, 706)	1.65	0.74	1.17
	p-NFH (592, 598, 604)	1.65	0.71	1.03
	p-NFH (730, 736, 742)	1.67	0.72	1.27
	p-NFH (512, 520)	1.68	0.67	0.82
	p-NFH (676, 679, 682)	1.70	0.73	1.02
	p-NFH (604, 610, 616)	1.70	0.76	1.05
	p-NFH (526, 532)	1.72	0.69	1.07
	p-NFH (556, 562)	1.78	0.77	1.05
	p-NFH (598, 604)	1.78	0.73	0.84
	p-NFH (661, 664, 670)	1.81	0.77	1.17
	p-NFH (652, 658)	1.82	0.57	0.55
	p-NFH (562, 568, 574)	1.84	0.66	1.02
	p-NFH (505)	1.96	0.83	1.27
	p-NFH (676, 682)	1.97	0.74	0.99
	p-NFH (532, 538)	2.00	0.53	0.60
	p-NFH (780)	2.01	0.76	1.23
	p-NFH (538, 544, 550)	2.02	0.63	0.75
	p-NFH (634, 643, 646)	2.05	0.69	3.38
	p-NFH (616, 619)	2.05	0.50	0.61
	p-NFH (619, 622, 628)	2.10	0.60	0.93
	p-NFH (568, 574, 583)	2.11	1.28	4.03
	p-NFH (652, 658, 664)	2.12	0.50	0.57
	p-NFH (664, 670)	2.15	0.50	0.66
	p-NFH (616, 622)	2.18	0.78	1.52
	p-NFH (610, 616)	2.19	0.48	0.49
	p-NFH (748)	2.35	0.81	1.43

	p-NFH (679, 682, 688)	2.36	0.73	0.78
	p-NFH (592, 598)	2.39	0.54	0.66
	p-NFH (688, 694)	2.41	0.76	1.71
	p-NFH (495, 505, 512)	2.56	0.58	0.51
	p-NFH (616, 622, 628)	2.58	0.61	1.45
	p-NFH (496, 505)	2.61	0.65	0.80
	p-NFH (544, 550, 556)	2.68	0.47	0.49
	p-NFH (433)	2.70	1.22	2.22
	p-NFH (616, 619, 622)	2.73	0.59	0.78
	p-NFH (497)	2.86	0.39	0.30
	p-NFH (700, 706, 712)	2.86	0.14	1.08
	p-NFH (844, 858)	3.05	1.10	5.41
	p-NFH (496)	3.10	0.47	0.58
	p-NFH (610, 616, 619)	3.17	0.37	0.37
	p-NFH (780, 794)	3.20	0.85	1.51
	p-NFH (544, 550)	3.29	0.47	0.39
	p-NFH (347)	3.37	0.35	0.46
	p-NFH (520, 526)	3.54	0.69	0.90
	p-NFH (670, 676, 679)	3.62	0.29	0.53
	p-NFH (619, 622)	3.71	0.42	0.40
	p-NFH (495, 505)	4.46	10.04	7.08
	p-NFH (676, 679, 688)	5.06	0.24	1.39
	p-NFH (700)	5.69	0.51	0.75
	p-NFH (574, 580, 586)	6.12	0.17	0.20
	p-NFH (670)	7.94	0.36	0.37
	p-NFH (640, 646, 652)	8.50	0.64	1.20
	p-NFH (497, 505, 510)	8.63	0.42	0.44
	p-NFH (550)	11.46	0.32	0.29
	p-NFH (676)	16.24	0.32	0.43
	p-MAP1B (526)	0.23	6.24	28.45
	p-MAP1B (1150)	0.33	2.20	3.78
	p-MAP1B (1199)	0.34	3.92	7.68
	p-MAP1B (1683)	0.40	2.06	9.03
	p-MAP1B (1646)	0.51	1.43	2.44
	p-MAP1B (1611)	0.58	0.77	0.95
	p-MAP1B (1371, 1382)	0.58	1.74	2.31
	p-MAP1B (1857, 1870)	0.59	5.42	0.49
	p-MAP1B (1254)	0.60	1.24	2.12
	p-MAP1B (1369)	0.60	1.50	1.61
	p-MAP1B (1865)	0.60	3.27	8.54
	p-MAP1B (1380)	0.63	1.43	1.56
	p-MAP1B (1786)	0.64	1.19	1.41
	p-MAP1B (1772)	0.70	1.37	2.00
	p-MAP1B (614)	0.70	1.35	1.04
	p-MAP1B (1432, 1436)	0.71	0.49	0.13
	p-MAP1B (336, 343)	0.72	1.28	0.08
	p-MAP1B (1786, 1789)	0.73	3.02	13.92
	p-MAP1B (1811)	0.74	1.26	2.02
	p-MAP1B (1812)	0.76	1.28	1.72
	p-MAP1B (825)	0.77	1.39	2.40
	p-MAP1B (2258)	0.79	1.30	1.47

	p-MAP1B (1382)	0.81	1.26	1.49
	p-MAP1B (561)	0.82	1.14	1.40
	p-MAP1B (1319, 1323)	0.83	1.42	0.67
	p-MAP1B (1619)	0.84	1.18	0.91
	p-MAP1B (1874)	0.84	1.02	1.04
	p-MAP1B (1872)	0.85	1.08	1.06
	p-MAP1B (988)	0.87	1.34	1.29
	p-MAP1B (1959)	0.87	0.94	1.57
	p-MAP1B (2027)	0.88	1.03	0.95
	p-MAP1B (2060)	0.89	0.70	0.94
	p-MAP1B (1908)	0.91	0.98	0.98
	p-MAP1B (1165)	0.91	1.25	1.81
	p-MAP1B (1175, 1185)	0.91	0.99	0.34
	p-MAP1B (1942)	0.92	1.00	1.28
	p-MAP1B (1248, 1254)	0.92	0.29	0.04
	p-MAP1B (1252, 1257)	0.93	1.04	1.72
	p-MAP1B (1033)	0.94	2.10	7.38
	p-MAP1B (1329)	0.94	0.68	0.58
	p-MAP1B (1772, 1778)	0.97	1.06	1.17
	p-MAP1B (1180)	0.99	1.01	0.81
	p-MAP1B (1870, 1872)	0.99	0.77	1.58
	p-MAP1B (541)	0.99	0.95	0.72
	p-MAP1B (1420)	1.01	0.85	1.85
	p-MAP1B (1925)	1.02	0.95	1.00
	p-MAP1B (2173)	1.02	0.84	2.31
	p-MAP1B (339)	1.03	0.96	0.76
	p-MAP1B (1494)	1.04	0.78	0.76
	p-MAP1B (1248, 1252)	1.05	0.82	1.41
MAP1B	p-MAP1B (544)	1.06	0.33	0.10
	p-MAP1B (1274)	1.07	1.10	1.30
	p-MAP1B (1317, 1329)	1.07	6.60	0.88
	p-MAP1B (1315)	1.08	0.92	0.05
	p-MAP1B (1401)	1.11	0.60	0.77
	p-MAP1B (1940)	1.12	0.72	1.57
	p-MAP1B (2054, 2065)	1.12	0.70	1.12
	p-MAP1B (1778)	1.16	0.87	0.71
	p-MAP1B (1436)	1.17	0.40	0.16
	p-MAP1B (1330)	1.19	0.86	1.93
	p-MAP1B (1611, 1613)	1.20	0.90	0.81
	p-MAP1B (965)	1.21	0.74	0.56
	p-MAP1B (1305)	1.23	0.73	1.60
	p-MAP1B (1239, 1244)	1.24	1.21	0.73
	p-MAP1B (1248, 1257)	1.24	0.43	0.52
	p-MAP1B (2059, 2065)	1.25	0.86	0.71
	p-MAP1B (985)	1.25	1.33	0.74
	p-MAP1B (1317)	1.26	0.94	1.50
	p-MAP1B (1239)	1.27	1.02	0.96
	p-MAP1B (1295)	1.28	0.76	1.65
	p-MAP1B (956, 965)	1.29	0.82	0.66
	p-MAP1B (2044)	1.30	1.00	2.07
	p-MAP1B (1244, 1248)	1.32	0.59	0.88
	p-MAP1B (1315, 1321)	1.37	0.89	0.79

	p-MAP1B (890)	1.37	0.76	0.74
	p-MAP1B (1659, 1683)	1.38	0.33	0.07
	p-MAP1B (1501)	1.39	0.85	1.88
	p-MAP1B (1244)	1.39	0.85	1.02
	p-MAP1B (963, 965)	1.40	0.74	0.69
	p-MAP1B (1772, 1775)	1.42	1.02	3.41
	p-MAP1B (884)	1.43	0.70	0.07
	p-MAP1B (1291)	1.47	0.63	1.12
	p-MAP1B (821, 824)	1.47	0.99	1.85
	p-MAP1B (1520)	1.52	0.68	0.71
	p-MAP1B (1009)	1.55	0.88	0.87
	p-MAP1B (1204)	1.55	0.93	0.61
	p-MAP1B (884, 901)	1.61	0.59	0.78
	p-MAP1B (1981)	1.80	0.56	0.08
	p-MAP1B (930)	1.82	0.70	0.75
	p-MAP1B (1659)	1.85	0.29	0.30
	p-MAP1B (2407)	1.91	0.51	0.10
	p-MAP1B (1525)	2.19	0.70	1.05
	p-MAP1B (892)	2.22	0.71	0.91
	p-MAP1B (884, 891)	2.30	0.68	0.77
	p-MAP1B (824, 825)	2.55	0.78	1.35
	p-MAP1B (958, 960)	2.63	0.67	0.85
	p-MAP1B (929)	2.86	0.61	0.53
	p-MAP1B (881, 884)	2.98	0.42	0.60
	p-MAP1B (941, 963)	3.00	0.78	1.54
	p-MAP1B (941)	4.64	0.50	0.22
	p-MAP1B (336, 339)	18.34	0.71	0.74
	p-ANK2 (31)	0.33	2.60	1.77
	p-ANK2 (2641;2745)	0.44	2.61	23.65
	p-ANK2 (UNK)	0.48	1.20	1.02
	p-ANK2 (3073;3177)	0.53	1.30	1.60
	p-ANK2 (UNK)	0.57	10.63	2.38
	p-ANK2 (3733;3837)	0.59	1.71	2.21
	p-ANK2 (1940;2044)	0.60	1.21	1.86
	p-ANK2 (2243;2347)	0.61	1.62	0.73
	p-ANK2 (2404;2508)	0.62	1.31	0.08
	p-ANK2 (1757;1861)	0.64	1.24	1.16
	p-ANK2 (1924;2028)	0.64	1.11	1.00
	p-ANK2 (1863;1967)	0.65	1.45	1.85
	p-ANK2 (2137;2241)	0.69	1.59	1.24
	p-ANK2 (1814;1918)	0.72	1.44	1.00
	p-ANK2 (3815;3919)	0.72	1.11	1.21
	p-ANK2 (3822;3926)	0.74	1.15	1.32
	p-ANK2 (2692;2796)	0.77	1.06	0.99
	p-ANK2 (31;48)	0.79	1.15	1.44
	p-ANK2 (2131;2235)	0.80	1.16	1.25
	p-ANK2 (2660;2764)	0.82	1.38	1.28
	p-ANK2 (3404;3508)	0.85	1.34	1.24
	p-ANK2 (1971;2075)	0.89	0.48	0.86
	p-ANK2 (2794;2898)	0.91	1.17	0.59
ANK2	p-ANK2 (3705;3809)	0.91	0.69	1.08

	p-ANK2 (UNK)	0.93	1.56	1.41
	p-ANK2 (3805;3909)	0.95	0.93	0.62
	p-ANK2 (3091;3195)	0.96	1.05	1.28
	p-ANK2 (3270;3374)	0.97	2.08	1.63
	p-ANK2 (2467;2571)	0.97	1.10	1.32
	p-ANK2 (1932;2036)	1.01	0.95	1.26
	p-ANK2 (1886;1990)	1.02	1.02	1.08
	p-ANK2 (UNK)	1.17	0.89	0.97
	p-ANK2 (3759;3863)	1.19	2.38	2.10
	p-ANK2 (3827;3931)	1.21	0.91	1.22
	p-ANK2 (2515;2619)	1.22	1.04	1.06
	p-ANK2 (3384;3488)	1.23	1.11	1.03
	p-ANK2 (3826;3930)	1.29	0.85	1.17
	p-ANK2 (3403)	1.38	0.53	0.89
	p-ANK2 (3895;3999)	1.48	0.54	0.77
	p-ANK2 (UNK)	1.65	0.87	1.43
	p-ANK2 (2471;2575)	1.70	0.84	0.07
	p-ANK2 (2243;2539)	2.06	0.77	0.29
	p-ANK2 (1734;1838)	2.14	1.15	0.99
	p-ANK2 (UNK)	2.63	1.93	5.54
	p-ANK2 (34;51)	3.06	0.77	0.80
	p-ANK2 (UNK)	11.53	0.19	0.46
TAU	p-TAU (58)	0.15	1.47	2.41
	p-TAU (57)	0.24	1.49	3.14
	p-TAU (UNK)	0.39	1.87	3.35
	p-TAU (UNK)	0.51	1.93	2.14
	p-TAU (UNK)	0.63	1.47	2.40
	p-TAU (UNK)	0.64	1.49	1.85
	p-TAU (UNK)	0.67	1.59	1.73
	p-TAU (UNK)	0.69	1.64	1.54
	p-TAU (UNK)	0.73	3.26	7.06
	p-TAU (UNK)	0.74	1.08	1.36
	p-TAU (UNK)	0.74	0.83	0.59
	p-TAU (191)	0.74	1.90	1.53
	p-TAU (UNK)	0.77	1.28	1.22
	p-TAU (UNK)	0.81	1.14	1.13
	p-TAU (UNK)	0.83	2.17	6.28
	p-TAU (UNK)	0.85	0.93	1.02
	p-TAU (UNK)	0.85	0.88	1.04
	p-TAU (UNK)	0.86	0.97	1.55
	p-TAU (UNK)	0.88	0.86	1.10
	p-TAU (UNK)	0.95	1.93	5.70
	p-TAU (UNK)	1.00	2.35	6.29
	p-TAU (UNK)	1.06	2.33	4.66
	p-TAU (UNK)	1.06	1.02	0.70
	p-TAU (UNK)	1.08	1.11	2.53
	p-TAU (UNK)	1.10	0.94	0.14
	p-TAU (UNK)	1.15	0.79	1.23
	p-TAU (50)	1.19	0.90	0.77
	p-TAU (UNK)	1.19	1.39	0.97
	p-TAU (UNK)	1.19	0.96	0.82

	p-TAU (58)	1.33	0.85	0.63
	p-TAU (UNK)	1.34	2.38	7.23
	p-TAU (UNK)	1.35	1.23	1.78
	p-TAU (60)	1.50	0.63	0.62
	p-TAU (71)	1.50	0.94	0.81
	p-TAU (204)	1.65	0.69	0.36
	p-TAU (278)	1.88	0.63	1.59
	p-TAU (UNK)	1.89	0.68	0.12
	p-TAU (50, 58)	1.97	0.75	0.59
	p-TAU (53)	2.13	0.59	0.30
	p-TAU (UNK)	2.91	0.50	0.30
EPB41L3	p-EPB41L3 (UNK)	0.49	3.01	3.90
	p-EPB41L3 (UNK)	0.55	1.50	1.39
	p-EPB41L3 (91)	0.59	0.35	0.11
	p-EPB41L3 (102)	0.59	1.60	2.63
	p-EPB41L3 (483)	0.67	1.15	0.14
	p-EPB41L3 (91, 94)	0.71	0.34	0.10
	p-EPB41L3 (UNK)	0.71	1.18	1.01
	p-EPB41L3 (UNK)	0.76	1.27	1.97
	p-EPB41L3 (538)	0.84	1.36	0.77
	p-EPB41L3 (UNK)	0.89	0.93	0.09
	p-EPB41L3 (423)	0.90	1.15	1.31
	p-EPB41L3 (UNK)	0.98	1.26	0.67
	p-EPB41L3 (UNK)	1.04	0.99	0.92
	p-EPB41L3 (446)	1.09	0.94	0.64
	p-EPB41L3 (UNK)	1.17	0.96	1.34
	p-EPB41L3 (UNK)	1.18	1.10	0.76
	p-EPB41L3 (66)	1.26	1.00	0.79
	p-EPB41L3 (5)	1.31	0.73	0.57
	p-EPB41L3 (UNK)	1.46	0.52	0.65
	p-EPB41L3 (UNK)	1.50	1.03	1.21
	p-EPB41L3 (UNK)	1.50	0.88	1.90
	p-EPB41L3 (11)	1.72	0.68	0.57
	p-EPB41L3 (453)	1.76	1.03	2.18
	p-EPB41L3 (UNK)	1.90	0.67	0.64
	p-EPB41L3 (UNK)	2.13	0.65	0.09
	p-EPB41L3 (407)	2.15	0.92	1.72
	p-EPB41L3 (UNK)	2.44	0.51	0.51
	p-EPB41L3 (UNK)	2.82	0.64	0.82
	p-EPB41L3 (UNK)	3.88	0.68	0.06
	p-EPB41L3 (UNK)	3.96	0.92	0.74
	p-EPB41L3 (UNK)	5.54	0.45	0.14
	p-PLAKOPHILIN 4 (UNK)	0.16	1.47	1.13
	p-PLAKOPHILIN 4 (UNK)	0.25	1.31	3.93
	p-PLAKOPHILIN 4 (UNK)	0.31	1.93	1.38
	p-PLAKOPHILIN 4 (UNK)	0.35	2.56	2.09
	p-PLAKOPHILIN 4 (UNK)	0.37	2.10	2.23
	p-PLAKOPHILIN 4 (UNK)	0.39	2.15	2.69
	p-PLAKOPHILIN 4 (UNK)	0.41	1.73	0.71
	p-PLAKOPHILIN 4 (UNK)	0.56	1.96	2.46
	p-PLAKOPHILIN 4 (UNK)	0.56	0.42	0.61

PLAKOPHILIN	p-PLAKOPHILIN 4 (UNK)	0.58	0.81	0.45
	p-PLAKOPHILIN 4 (UNK)	0.59	2.06	1.94
	p-PLAKOPHILIN 4 (UNK)	0.68	1.76	0.39
	p-PLAKOPHILIN 4 (UNK)	0.71	1.10	1.42
	p-PLAKOPHILIN 4 (UNK)	0.71	1.42	2.24
	p-PLAKOPHILIN 4 (UNK)	0.79	1.49	1.48
	p-PLAKOPHILIN 4 (UNK)	0.79	0.47	0.47
	p-PLAKOPHILIN 4 (UNK)	0.81	1.18	1.62
	p-PLAKOPHILIN 4 (143)	0.82	1.06	1.31
	p-PLAKOPHILIN 4 (UNK)	0.87	1.11	0.90
	p-PLAKOPHILIN 4 (UNK)	0.91	0.94	5.40
	p-PLAKOPHILIN 4 (UNK)	0.92	1.43	0.34
	p-PLAKOPHILIN 4 (UNK)	0.94	1.25	1.74
	p-PLAKOPHILIN 4 (UNK)	0.98	1.32	1.04
	p-PLAKOPHILIN 4 (UNK)	1.03	1.36	1.01
	p-PLAKOPHILIN 4 (UNK)	1.04	0.92	0.50
	p-PLAKOPHILIN 4 (UNK)	1.08	0.79	1.44
	p-PLAKOPHILIN 4 (UNK)	1.19	1.22	0.65
TENSIN 1	p-TENSIN 1 (971)	0.41	2.32	5.04
	p-TENSIN 1 (1587;1566)	0.49	1.54	0.34
	p-TENSIN 1 (UNK)	0.55	1.25	3.77
	p-TENSIN 1 (1418;1397)	0.83	0.70	0.36
	p-TENSIN 1 (870)	0.85	0.73	0.42
	p-TENSIN 1 (927)	0.89	0.85	0.70
	p-TENSIN 1 (1482;1461)	0.90	1.12	1.65
	p-TENSIN 1 (648)	0.96	2.24	10.25
	p-TENSIN 1 (606)	1.03	0.71	0.27
	p-TENSIN 1 (754)	1.08	0.88	1.13
	p-TENSIN 1 (949)	1.09	1.25	2.00
	p-TENSIN 1 (547)	1.12	0.50	0.94
	p-TENSIN 1 (1362;1341)	1.19	0.77	0.91
	p-TENSIN 1 (918)	1.22	0.73	0.52
	p-TENSIN 1 (1554;1533)	1.24	0.16	0.06
	p-TENSIN 1 (1145)	1.25	0.90	1.32
	p-TENSIN 1 (1566;1545)	1.28	0.64	1.33
	p-TENSIN 1 (951)	1.58	0.90	1.79
	p-TENSIN 1 (1034)	1.66	0.77	0.83
	p-TENSIN 1 (1032)	1.66	0.77	0.04
	p-TENSIN 1 (1487;1466)	1.90	0.81	1.03
	p-TENSIN 1 (1573;1552)	2.27	0.34	1.21
	p-TENSIN 1 (511)	2.65	0.52	0.92
	p-TENSIN 1 (1073)	2.76	0.49	0.92
	p-TENSIN 1 (794)	2.85	0.83	1.26
	p-NFL (519, 522)	0.51	1.29	1.02
	p-NFL (50)	0.64	2.09	1.49
	p-NFL (52)	0.74	1.37	2.17
	p-NFL (291)	0.94	0.75	1.09
	p-NFL (436)	0.98	1.69	0.71
	p-NFL (430)	0.98	1.01	0.89
	p-NFL (48)	0.99	2.16	1.56
	p-NFL (317)	0.99	1.17	0.91

NFL	p-NFL (41)	1.03	1.43	0.81
	p-NFL (27)	1.21	0.84	0.82
	p-NFL (42)	1.28	0.58	1.19
	p-NFL (283)	1.31	0.99	1.15
	p-NFL (184)	1.36	0.69	1.05
	p-NFL (425)	1.38	0.98	1.26
	p-NFL (44)	1.39	1.21	0.82
	p-NFL (103)	1.47	0.77	0.69
	p-NFL (473)	1.86	0.90	0.26
	p-NFL (444, 473)	2.08	0.33	1.98
	p-NFL (426)	2.09	0.83	1.26
	p-NFL (34)	2.32	0.44	2.76
	p-NFL (473, 503)	3.18	1.32	0.56
	p-NFL (519, 531)	3.28	0.63	0.50
	p-NFL (462)	4.49	0.23	0.69
	p-NFL (522, 531)	6.60	0.60	0.51
	p-NFL (3)	7.51	0.74	40.94
BSN	p-BSN (142)	0.44	1.42	0.92
	p-BSN (2033)	0.59	1.36	1.76
	p-BSN (2844, 2850)	0.59	1.60	1.70
	p-BSN (1930)	0.60	1.25	1.22
	p-BSN (3501)	0.60	1.34	1.82
	p-BSN (3006)	0.61	1.71	1.43
	p-BSN (1154)	0.63	1.59	0.70
	p-BSN (148)	0.64	1.19	0.69
	p-BSN (1468, 1484)	0.65	6.77	5.19
	p-BSN (2850)	0.65	1.55	1.31
	p-BSN (1086, 1089, 1098)	0.67	1.65	2.47
	p-BSN (2055)	0.70	1.76	1.57
	p-BSN (2844)	0.73	1.27	1.31
	p-BSN (105)	0.77	0.26	0.12
	p-BSN (1468)	0.78	2.70	4.48
	p-BSN (1478)	0.79	1.21	1.53
	p-BSN (1469)	0.88	0.95	0.36
	p-BSN (2607)	0.89	0.88	0.78
	p-BSN (1002)	0.91	1.06	0.59
	p-BSN (3367)	0.94	1.20	1.14
	p-BSN (2580)	1.24	1.32	1.08
	p-BSN (717)	1.45	0.83	0.29
	p-BSN (964)	1.59	0.52	0.58
	p-SYN1 (11)	0.22	1.28	3.21
	p-SYN1 (508)	0.37	1.75	1.54
	p-SYN1 (434)	0.39	11.70	24.67
	p-SYN1 (680)	0.46	1.59	1.52
	p-SYN1 (510)	0.48	0.72	0.99
	p-SYN1 (9)	0.50	0.85	1.12
	p-SYN1 (666)	0.54	1.46	1.41
	p-SYN1 (664, 680)	0.55	1.91	0.40
	p-SYN1 (62)	0.57	1.17	2.47
	p-SYN1 (664)	0.61	1.98	2.69
	p-SYN1 (549)	0.71	0.57	1.30

SYN1	p-SYN1 (432, 436)	0.76	1.29	1.47
	p-SYN1 (436)	0.92	1.14	0.82
	p-SYN1 (425, 436)	0.99	1.04	0.93
	p-SYN1 (425)	1.04	1.04	0.66
	p-SYN1 (430)	1.05	0.74	1.55
	p-SYN1 (332)	1.11	1.19	0.92
	p-SYN1 (566)	1.21	0.59	0.48
	p-SYN1 (430, 436)	1.21	0.93	1.21
	p-SYN1 (549, 551)	1.48	0.13	0.16
	p-SYN1 (71)	1.67	1.23	1.58
	p-SYN1 (579)	2.66	1.95	1.14
	p-SYN1 (603)	3.43	0.70	0.23
	p-PICCOLO (1304)	0.51	0.68	1.49
PICCOLO	p-PICCOLO (1841)	0.56	1.52	2.17
	p-PICCOLO (1840)	0.57	2.07	2.47
	p-PICCOLO (814)	0.60	3.72	0.70
	p-PICCOLO (1812)	0.69	1.59	1.61
	p-PICCOLO (1575)	0.74	1.31	0.85
	p-PICCOLO (1820)	0.84	1.34	1.76
	p-PICCOLO (1451)	0.84	1.13	1.03
	p-PICCOLO (3781)	0.85	1.06	0.86
	p-PICCOLO (4343)	0.86	1.17	0.82
	p-PICCOLO (1353)	0.90	1.21	1.06
	p-PICCOLO (4340, 4341)	0.93	1.35	1.87
	p-PICCOLO (1587)	0.93	1.21	0.73
	p-PICCOLO (1772, 1775, 1784)	0.97	0.92	2.01
	p-PICCOLO (1352)	0.97	1.69	0.21
	p-PICCOLO (4150)	1.03	1.02	1.02
	p-PICCOLO (4723)	1.03	1.58	1.28
	p-PICCOLO (3779)	1.09	0.92	0.86
	p-PICCOLO (1772, 1778, 1784)	1.11	0.95	1.42
	p-PICCOLO (4801)	1.19	2.48	0.83
	p-PICCOLO (3614)	1.30	1.22	0.40
	p-PICCOLO (1464)	1.37	1.10	0.76
	p-PICCOLO (1677)	1.58	0.94	0.24
CAMK2B	p-CAMK2B (unk)	0.35	3.21	2.39
	p-CAMK2B (UNK)	0.47	1.65	3.50
	p-CAMK2B (UNK)	0.70	0.90	0.71
	p-CAMK2B (367)	0.75	1.28	1.11
	p-CAMK2B (UNK)	0.86	0.64	1.40
	p-CAMK2B (UNK)	0.86	0.46	0.87
	p-CAMK2B (280)	0.89	1.26	1.35
	p-CAMK2B (UNK)	0.89	2.29	0.67
	p-CAMK2B (UNK)	0.92	0.72	0.75
	p-CAMK2B (UNK)	0.92	1.38	1.43
	p-CAMK2B (UNK)	0.99	1.11	0.85
	p-CAMK2B (UNK)	1.05	0.82	0.76
	p-CAMK2B (UNK)	1.13	0.65	0.39
	p-CAMK2B (UNK)	1.15	0.54	0.60
	p-CAMK2B (277)	1.23	1.73	1.32
	p-CAMK2B (287)	1.25	1.12	1.39

	p-CAMK2B (UNK)	1.77	0.59	0.67
	p-CAMK2B (276)	2.22	1.00	0.84
	p-CAMK2B (UNK)	2.96	0.67	0.82
PLECTIN 1	p-PLECTIN-1 (UNK)	0.63	2.12	2.67
	p-PLECTIN-1 (UNK)	0.80	0.87	1.12
	p-PLECTIN-1 (UNK)	0.89	1.10	1.19
	p-PLECTIN-1 (UNK)	0.90	0.13	0.53
	p-PLECTIN-1 (UNK)	1.05	0.85	1.03
	p-PLECTIN-1 (UNK)	1.14	0.83	1.22
	p-PLECTIN-1 (UNK)	1.17	0.63	0.53
	p-PLECTIN-1 (UNK)	1.20	0.55	0.68
	p-PLECTIN-1 (UNK)	1.21	0.88	1.07
	p-PLECTIN-1 (UNK)	1.25	0.82	1.53
	p-PLECTIN-1 (UNK)	1.52	0.42	0.33
	p-PLECTIN-1 (UNK)	2.90	1.04	4.70
	p-PLECTIN-1 (21)	3.66	0.45	1.83
	p-PLECTIN-1 (UNK)	4.12	0.84	2.27
	p-PLECTIN-1 (UNK)	5.12	0.61	1.42
	p-PLECTIN-1 (UNK)	6.06	0.24	0.88
	p-PLECTIN-1 (UNK)	13.61	0.24	1.27
NDRG1	p-NDRG1 (375)	0.52	1.01	0.87
	p-NDRG1 (330, 333)	0.74	0.73	0.80
	p-NDRG1 (333, 336)	0.90	0.42	0.89
	p-NDRG1 (367)	1.05	0.91	1.01
	p-NDRG1 (333)	1.15	0.55	0.48
	p-NDRG1 (330)	1.26	0.52	0.51
	p-NDRG1 (332, 333)	1.30	0.51	0.40
	p-NDRG1 (319)	1.53	0.47	0.69
	p-NDRG1 (332)	1.53	0.43	0.43
	p-NDRG1 (328, 332, 336)	1.57	0.31	0.31
	p-NDRG1 (366)	1.63	0.78	0.92
	p-NDRG1 (328, 330)	2.02	0.67	0.87
	p-NDRG1 (342)	2.32	1.21	1.17
	p-NDRG1 (330, 333, 336)	2.96	0.37	1.75
	p-NDRG1 (362, 364)	3.23	0.52	0.45
RIMS1	p-RIMS1 (UNK)	0.33	2.18	1.79
	p-RIMS1 (UNK)	0.33	2.96	3.68
	p-RIMS1 (UNK)	0.50	1.55	0.10
	p-RIMS1 (UNK)	0.54	1.93	1.03
	p-RIMS1 (UNK)	0.57	0.89	0.66
	p-RIMS1 (UNK)	0.60	1.28	1.57
	p-RIMS1 (UNK)	0.61	1.15	1.87
	p-RIMS1 (UNK)	0.74	1.13	0.83
	p-RIMS1 (UNK)	0.78	1.56	1.25
	p-RIMS1 (UNK)	0.83	1.36	0.94
	p-RIMS1 (UNK)	0.86	0.61	0.52
	p-RIMS1 (UNK)	0.89	1.25	1.35
	p-RIMS1 (UNK)	1.06	0.99	1.43
	p-RIMS1 (UNK)	1.55	1.34	2.88
	p-GEPHYRIN (188, 194)	0.20	1.61	5.19
	p-GEPHYRIN (283)	0.50	1.05	0.67

	p-GEPHYRIN (319)	0.62	1.22	1.16
	p-GEPHYRIN (286)	0.73	1.20	0.76
	p-GEPHYRIN (266)	0.81	1.09	1.22
	p-GEPHYRIN (305)	0.87	1.16	1.19
	p-GEPHYRIN (264, 270)	0.88	1.02	1.06
GEPHYRIN	p-GEPHYRIN (270)	0.92	1.16	0.90
	p-GEPHYRIN (265, 270)	0.95	1.05	0.94
	p-GEPHYRIN (194)	1.03	1.02	0.79
	p-GEPHYRIN (188, 200)	1.05	0.61	0.60
	p-GEPHYRIN (265)	1.13	1.13	0.71
	p-GEPHYRIN (188, 198)	1.39	0.83	0.56
	p-GEPHYRIN (188)	1.49	0.89	0.43
	p-ZO1 (UNK)	0.56	1.45	1.61
	p-ZO1 (UNK)	0.73	1.67	1.23
	p-ZO1 (UNK)	0.92	0.52	1.62
	p-ZO1 (UNK)	1.00	1.10	0.70
	p-ZO1 (UNK)	1.10	1.25	0.58
	p-ZO1 (UNK)	1.12	0.59	0.52
ZO1	p-ZO1 (UNK)	1.15	0.46	0.37
	p-ZO1 (UNK)	1.43	0.90	0.67
	p-ZO1 (UNK)	1.75	0.69	0.85
	p-ZO1 (UNK)	1.82	0.74	4.76
	p-ZO1 (UNK)	2.18	0.58	0.79
	p-ZO1 (UNK)	4.42	0.57	0.92
	p-CAMSAP3 (UNK)	0.24	2.68	3.38
	p-CAMSAP3 (384)	0.37	1.21	0.95
	p-CAMSAP3 (UNK)	0.42	1.63	1.27
	p-CAMSAP3 (UNK)	0.43	1.71	0.27
	p-CAMSAP3 (UNK)	0.55	1.02	2.87
CAMSAP3	p-CAMSAP3 (UNK)	0.68	1.28	0.94
	p-CAMSAP3 (UNK)	0.68	1.20	1.35
	p-CAMSAP3 (UNK)	0.76	1.29	1.03
	p-CAMSAP3 (363)	0.78	1.22	0.68
	p-CAMSAP3 (UNK)	1.10	0.90	1.23
	p-CAMSAP3 (UNK)	1.10	0.52	0.58
	p-PARALEMMIN (145)	0.39	1.71	2.92
	p-PARALEMMIN (141)	0.45	1.84	4.53
	p-PARALEMMIN (363)	0.46	1.29	1.28
	p-PARALEMMIN (157)	0.48	6.33	3.38
	p-PARALEMMIN (116, 124)	0.56	1.61	1.48
PARALEMMIN	p-PARALEMMIN (153, 157)	0.72	1.79	1.38
	p-PARALEMMIN (153)	0.91	1.32	1.00
	p-PARALEMMIN (124)	0.99	1.04	0.86
	p-PARALEMMIN (122)	1.01	1.04	0.16
	p-PARALEMMIN (265)	1.01	1.30	1.15
	p-PARALEMMIN (123, 124)	1.18	0.90	0.99
	p-CTNND2 (UNK)	0.25	2.25	1.62
	p-CTNND2 (UNK)	0.30	2.47	2.33
	p-CTNND2 (398;412)	0.41	2.04	3.06
	p-CTNND2 (UNK)	0.47	1.31	1.35
CTNND2	p-CTNND2 (UNK)	0.73	1.20	1.38

CTNND2	p-CTNND2 (UNK)	0.84	1.17	0.94
	p-CTNND2 (UNK)	1.02	0.67	0.65
	p-CTNND2 (32)	1.18	0.67	2.36
	p-CTNND2 (UNK)	2.07	0.83	0.49
	p-CTNND2 (UNK)	3.74	1.34	1.92
CACNA1A	p-CACNA1A (752)	0.42	1.46	2.17
	p-CACNA1A (UNK)	0.46	0.57	0.46
	p-CACNA1A (UNK)	0.49	1.76	0.49
	p-CACNA1A (2071)	0.62	0.92	1.05
	p-CACNA1A (UNK)	0.78	3.92	1.84
	p-CACNA1A (792)	1.01	0.15	0.13
	p-CACNA1A (2028)	1.06	1.10	0.75
	p-CACNA1A (UNK)	1.39	1.47	1.24
	p-CACNA1A (UNK)	1.40	0.82	0.76
	p-CACNA1A (2068)	1.45	0.36	0.08
ANK3	p-ANK3 iso2 (1854)	0.11	3.89	10.94
	p-ANK3 iso2 (631;606)	0.49	1.79	2.34
	p-ANK3 iso2 (2457)	0.64	1.15	1.34
	p-ANK3 iso2 (2102)	0.85	0.42	0.79
	p-ANK3 iso2 (1458)	0.88	1.36	2.31
	p-ANK3 iso2 (1984)	0.97	1.54	1.19
	p-ANK3 iso2 (2247)	1.84	0.40	0.72
	p-ANK3 iso2 (869, 875)	2.47	0.33	0.44
	p-ANK3 iso2 (875)	3.06	0.59	0.54
MYELIN P0	p-MYELIN P0 (226)	0.59	0.56	0.14
	p-MYELIN P0 (195)	0.81	0.99	1.55
	p-MYELIN P0 (216)	0.81	0.85	0.67
	p-MYELIN P0 (294)	1.32	0.71	1.10
	p-MYELIN P0 (292)	1.60	0.45	0.87
	p-MYELIN P0 (294, 297)	6.52	0.65	1.47
	p-MYELIN P0 (78)	7.01	0.31	0.46
	p-MYELIN P0 (106)	11.19	0.21	0.43
CTNND1	p-CTNND1 (UNK)	0.36	3.58	4.18
	p-CTNND1 (349)	0.75	1.07	1.12
	p-CTNND1 (47)	1.06	0.64	0.14
	p-CTNND1 (288)	1.33	0.56	1.11
	p-CTNND1 (252)	1.58	1.16	2.71
	p-CTNND1 (228)	1.89	0.60	0.78
	p-CTNND1 (352)	1.98	0.77	1.44
	p-CTNND1 (268)	17.92	0.40	1.24
MGLUR5	p-MGLUR5 (860)	0.09	7.29	6.76
	p-MGLUR5 (901;933)	0.18	8.26	12.33
	p-MGLUR5 (839)	0.35	1.65	0.10
	p-MGLUR5 (1141, 1185)	0.38	1.41	1.60
	p-MGLUR5 (1152;1185)	0.63	1.04	0.63
	p-MGLUR5 (1016)	0.65	1.75	1.79
	p-MGLUR5 (1170;1202)	0.87	1.67	1.25
IQSEC3	p-IQSEC3 (641;342)	0.43	2.06	2.81
	p-IQSEC3 (1066, 770)	0.58	0.72	1.31
	p-IQSEC3 (131)	0.72	1.44	0.98
	p-IQSEC3 (644;345)	0.78	1.08	0.59

	p-IQSEC3 (1151;852)	0.86	0.64	1.17
	p-IQSEC3 (255)	0.89	1.00	1.02
	p-IQSEC3 (1053;754)	1.35	1.16	1.19
SPARCL1	p-SPARCL1 (340)	0.49	3.03	4.68
	p-SPARCL1 (68)	0.74	1.19	0.87
	p-SPARCL1 (68, 76)	0.81	1.32	1.10
	p-SPARCL1 (76)	0.82	1.15	0.80
	p-SPARCL1 (333, 340)	0.95	0.99	0.67
	p-SPARCL1 (68, 77)	1.02	1.23	0.96
	p-SPARCL1 (151)	1.22	1.04	0.30
NCAM-L1	p-NCAM-L1 iso2 (1173, 1180)	0.91	0.37	1.70
	p-NCAM-L1 iso2 (1175;1180)	1.02	1.20	0.63
	p-NCAM-L1 iso2 (1173;1178)	1.06	1.01	0.58
	p-NCAM-L1 iso2 (1178;1183)	1.07	0.92	1.60
	p-NCAM-L1 iso2 (1160, 1174)	1.10	0.85	1.14
	p-NCAM-L1 iso2 (UNK)	1.21	0.93	0.77
	p-NCAM-L1 iso2 (1173, 1183)	1.66	0.86	0.85
MYH10	p-MYH10 (1952)	0.88	0.70	0.95
	p-MYH10 (214)	0.96	0.57	0.42
	p-MYH10 (1954)	0.98	1.10	0.89
	p-MYH10 (UNK)	1.40	0.72	0.97
	p-MYH10 (1975)	1.42	1.20	3.13
	p-MYH10 (1956)	2.17	0.37	0.11
INA	p-INA (30)	0.50	2.56	2.43
	p-INA (37)	0.83	1.34	2.51
	p-INA (335)	0.89	1.14	1.35
	p-INA (441)	1.35	0.61	0.99
	p-INA (433)	4.27	0.63	0.40
ITGB4	p-ITGB4 (1405)	1.12	0.82	1.31
	p-ITGB4 (1121)	1.37	0.68	1.36
	p-ITGB4 (1366)	2.72	0.56	2.34
	p-ITGB4 (1776)	3.61	0.34	1.36
	p-ITGB4 (1389)	9.06	0.73	2.90
ACTG1	p-ACTG1 (240;241;242)	0.61	1.21	1.34
	p-ACTG1 (60;61;62)	0.82	0.81	1.20
	p-ACTG1 (203)	1.65	0.87	0.77
	p-ACTG1 (UNK)	1.89	0.81	0.95
	p-ACTG1 (199)	3.98	0.34	0.44
SAPAP3	p-SAPAP3 (750)	0.16	6.44	0.49
	p-SAPAP3 (185)	0.59	2.03	2.34
	p-SAPAP3 (58)	0.80	1.13	0.22
	p-SAPAP3 (712)	1.14	1.30	1.06
	p-SAPAP3 (965)	3.79	1.72	0.30
UNC13C	p-UNC13C (450)	0.65	1.79	1.21
	p-UNC13C (89)	0.68	1.33	1.19
	p-UNC13C (570)	0.93	1.94	1.36
	p-UNC13C (883)	1.37	0.69	0.85
	p-UNC13C (537)	6.28	0.29	0.12
RABPHILIN 3A	p-RABPHILIN 3A (683)	0.71	1.64	1.66
	p-RABPHILIN 3A (281;284)	0.94	1.05	1.50
	p-RABPHILIN 3A (682)	0.96	1.31	1.42

	p-RABPHILIN 3A (259)	1.18	0.61	0.63
	p-RABPHILIN 3A (262)	1.41	0.72	0.41
FLNA	p-FLNA (2172;2180)	0.90	1.07	1.57
	p-FLNA (UNK)	1.08	0.69	1.35
	p-FLNA (968)	1.65	0.71	0.91
	p-FLNA (1459)	2.30	0.34	0.29
	p-FLNA (2144;2152)	2.40	0.50	0.69
PPFIA3	p-PPFIA3 (1162;1153)	0.16	3.32	8.32
	p-PPFIA3 (142)	0.85	1.01	1.11
	p-PPFIA3 (667)	0.85	2.26	0.41
	p-PPFIA3 (712)	1.27	0.59	1.00
RIMS2	p-RIMS2 (UNK)	0.48	2.22	4.02
	p-RIMS2 (UNK)	0.66	1.19	1.05
	p-RIMS2 (UNK)	0.78	0.74	0.23
	p-RIMS2 (UNK)	1.46	1.42	1.65
ATP2B2	p-ATP2B2 (1117)	0.16	6.09	11.60
	p-ATP2B2 (1118)	0.37	2.47	4.26
	p-ATP2B2 (18)	1.07	0.90	0.87
	p-ATP2B2 (27)	1.11	0.88	0.88
SLC6A1	p-SLC6A1 (14)	0.86	1.30	0.13
	p-SLC6A1 (15)	0.87	0.98	0.65
	p-SLC6A1 (18)	1.01	0.98	0.91
	p-SLC6A1 (14, 18)	1.23	1.02	0.88
PRR14	p-PRR14 (80)	0.73	0.83	0.73
	p-PRR14 (164;71)	0.86	1.40	1.06
	p-PRR14 (78)	1.06	1.63	1.24
	p-PRR14 (158;65)	1.32	1.01	1.09
CACNB4	p-CACNB4 (UNK)	0.44	1.78	1.83
	p-CACNB4 (377)	0.88	1.38	0.72
	p-CACNB4 (UNK)	2.53	0.74	0.58
DRP2	p-DRP2 (UNK)	1.47	0.74	1.05
	p-DRP2 (UNK)	2.56	0.46	0.53
	p-DRP2 (UNK)	6.34	0.44	1.03
DYN3	p-DYN3 (UNK)	0.28	2.08	3.79
	p-DYN3 (UNK)	0.29	4.29	11.57
	p-DYN3 (UNK)	1.10	1.15	0.39
SPTBN2	p-SPTBN2 (2254)	0.65	1.27	1.34
	p-SPTBN2 (2199)	0.90	0.97	0.97
	p-SPTBN2 (2200;2207)	1.10	0.90	0.79
UTROPHIN	p-UTROPHIN (10)	0.58	1.04	0.85
	p-UTROPHIN (UNK)	1.83	0.27	0.07
	p-UTROPHIN (UNK)	3.52	0.36	0.32
NEURABIN 1	p-NEURABIN 1 (192)	0.32	0.43	2.94
	p-NEURABIN 1 (UNK)	0.62	0.87	1.34
	p-NEURABIN 1 (372)	0.87	1.07	0.78
BCAN	p-BCAN (546)	0.73	0.54	1.00
	p-BCAN (406)	1.16	1.02	1.86
	p-BCAN (29)	1.25	0.95	0.84
MYO5A	p-MYO5A (600)	1.07	0.96	1.08
	p-MYO5A (1624)	1.56	0.61	0.13
	p-MYO5A (1115)	1.77	0.71	0.77

DBNL	p-DBNL (24)	0.92	0.93	0.90
	p-DBNL (311)	1.03	0.39	0.55
	p-DBNL (291)	1.65	0.49	0.73
VINCULIN	p-VINCULIN (721)	1.54	0.63	0.71
	p-VINCULIN (346)	1.61	0.78	0.84
TALIN1	p-TALIN 1 (1260;1277)	1.47	0.52	0.67
	p-TALIN 1 (425)	2.24	0.74	1.07
PSD-95	p-PSD-95 (UNK)	0.77	1.16	1.44
	p-PSD-95 (415)	1.31	1.03	0.60
LIPRIN alpha 4	p-LIPRIN alpha 4 (642)	0.55	1.62	1.14
	p-LIPRIN alpha 4 (614)	0.91	0.68	1.19
APOE	p-APOE (139)	2.50	0.60	0.64
CHAT	p-CHAT (365)	1.61	0.41	0.59
ESAM	p-ESAM (370)	1.26	0.81	0.61
FGF13	p-FGF13 (UNK)	0.87	1.40	1.26
PXN	p-PXN (UNK)	1.50	0.51	0.67
RAB3A	p-RAB3A (63)	1.69	0.51	0.70
SLC8A3	p-SLC8A3 (634)	0.64	1.20	1.86
TNC	p-TNC (72)	3.56	0.47	0.07
CADM1	p-CADM1 (468)	1.32	0.77	0.93
CTNNA1	p-CTNNA1 (643)	1.18	0.70	0.90
HAPLN4	p-HAPLN4 (195)	0.86	1.63	0.09
PDK1	p-PDK1 (244;217)	0.89	1.20	0.90
WAVE1	p-WAVE1 (310)	0.49	0.72	1.36
CNTNAP	p-CNTNAP (1380)	1.12	1.14	1.00
ERC2	p-ERC2 (910)	0.78	0.86	0.59
WASP	p-N-WASP (253)	1.01	1.14	1.83

Numbers in parenthesis indicate phosphorylated residues. Comma separation indicates multiple phosphorylation. Semicolon separation indicates possible residue location
