# Supplementary material for: Regulation of Expression of Extracellular Matrix Proteins by Differential Target Multiplexed Spinal Cord Stimulation (SCS) and Traditional Low-Rate SCS in a Rat Nerve Injury Model
Source: Biology (Basel). 2023 Mar 31;12(4):537. doi: 10.3390/biology12040537 (PMC10135794; doi:10.3390/biology12040537)
Supplement: Supplementary file 1 [file biology-12-00537-s001.zip › TableS7.pdf]

Table S7. Cell Signaling ECM Phosphoproteins - Fold Changes

Protein	Phosphoprotein Isoform	No-SCS / No-SNI	DTMP / No-SCS	LR-SCS / No-SCS
ANK2	p-ANK2 (31)	0.33	2.60	1.77
	p-ANK2 (2641;2745)	0.44	2.61	23.65
	p-ANK2 (UNK)	0.48	1.20	1.02
	p-ANK2 (3073;3177)	0.53	1.30	1.60
	p-ANK2 (UNK)	0.57	10.63	2.38
	p-ANK2 (3733;3837)	0.59	1.71	2.21
	p-ANK2 (1940;2044)	0.60	1.21	1.86
	p-ANK2 (2243;2347)	0.61	1.62	0.73
	p-ANK2 (2404;2508)	0.62	1.31	0.08
	p-ANK2 (1757;1861)	0.64	1.24	1.16
	p-ANK2 (1924;2028)	0.64	1.11	1.00
	p-ANK2 (1863;1967)	0.65	1.45	1.85
	p-ANK2 (2137;2241)	0.69	1.59	1.24
	p-ANK2 (1814;1918)	0.72	1.44	1.00
	p-ANK2 (3815;3919)	0.72	1.11	1.21
	p-ANK2 (3822;3926)	0.74	1.15	1.32
	p-ANK2 (2692;2796)	0.77	1.06	0.99
	p-ANK2 (31;48)	0.79	1.15	1.44
	p-ANK2 (2131;2235)	0.80	1.16	1.25
	p-ANK2 (2660;2764)	0.82	1.38	1.28
	p-ANK2 (3404;3508)	0.85	1.34	1.24
	p-ANK2 (1971;2075)	0.89	0.48	0.86
	p-ANK2 (2794;2898)	0.91	1.17	0.59
	p-ANK2 (3705;3809)	0.91	0.69	1.08
	p-ANK2 (UNK)	0.93	1.56	1.41
	p-ANK2 (3805;3909)	0.95	0.93	0.62
	p-ANK2 (3091;3195)	0.96	1.05	1.28
	p-ANK2 (3270;3374)	0.97	2.08	1.63
	p-ANK2 (2467;2571)	0.97	1.10	1.32
	p-ANK2 (1932;2036)	1.01	0.95	1.26
	p-ANK2 (1886;1990)	1.02	1.02	1.08
	p-ANK2 (UNK)	1.17	0.89	0.97
	p-ANK2 (3759;3863)	1.19	2.38	2.10
	p-ANK2 (3827;3931)	1.21	0.91	1.22
	p-ANK2 (2515;2619)	1.22	1.04	1.06
	p-ANK2 (3384;3488)	1.23	1.11	1.03
	p-ANK2 (3826;3930)	1.29	0.85	1.17
	p-ANK2 (3403)	1.38	0.53	0.89
	p-ANK2 (3895;3999)	1.48	0.54	0.77
	p-ANK2 (UNK)	1.65	0.87	1.43
	p-ANK2 (2471;2575)	1.70	0.84	0.07
	p-ANK2 (2243;2539)	2.06	0.77	0.29
	p-ANK2 (1734;1838)	2.14	1.15	0.99
	p-ANK2 (UNK)	2.63	1.93	5.54
	p-ANK2 (34;51)	3.06	0.77	0.80
	p-ANK2 (UNK)	11.53	0.19	0.46
	p-TAU (58)	0.15	1.47	2.41

	p-TAU (57)	0.24	1.49	3.14
	p-TAU (UNK)	0.39	1.87	3.35
	p-TAU (UNK)	0.51	1.93	2.14
	p-TAU (UNK)	0.63	1.47	2.40
	p-TAU (UNK)	0.64	1.49	1.85
	p-TAU (UNK)	0.67	1.59	1.73
	p-TAU (UNK)	0.69	1.64	1.54
	p-TAU (UNK)	0.73	3.26	7.06
	p-TAU (UNK)	0.74	1.08	1.36
	p-TAU (UNK)	0.74	0.83	0.59
	p-TAU (191)	0.74	1.90	1.53
	p-TAU (UNK)	0.77	1.28	1.22
	p-TAU (UNK)	0.81	1.14	1.13
	p-TAU (UNK)	0.83	2.17	6.28
	p-TAU (UNK)	0.85	0.93	1.02
	p-TAU (UNK)	0.85	0.88	1.04
	p-TAU (UNK)	0.86	0.97	1.55
	p-TAU (UNK)	0.88	0.86	1.10
TAU	p-TAU (UNK)	0.95	1.93	5.70
	p-TAU (UNK)	1.00	2.35	6.29
	p-TAU (UNK)	1.06	2.33	4.66
	p-TAU (UNK)	1.06	1.02	0.70
	p-TAU (UNK)	1.08	1.11	2.53
	p-TAU (UNK)	1.10	0.94	0.14
	p-TAU (UNK)	1.15	0.79	1.23
	p-TAU (50)	1.19	0.90	0.77
	p-TAU (UNK)	1.19	1.39	0.97
	p-TAU (UNK)	1.19	0.96	0.82
	p-TAU (58)	1.33	0.85	0.63
	p-TAU (UNK)	1.34	2.38	7.23
	p-TAU (UNK)	1.35	1.23	1.78
	p-TAU (60)	1.50	0.63	0.62
	p-TAU (71)	1.50	0.94	0.81
	p-TAU (204)	1.65	0.69	0.36
	p-TAU (278)	1.88	0.63	1.59
	p-TAU (UNK)	1.89	0.68	0.12
	p-TAU (50, 58)	1.97	0.75	0.59
	p-TAU (53)	2.13	0.59	0.30
	p-TAU (UNK)	2.91	0.50	0.30
	p-BSN (142)	0.44	1.42	0.92
	p-BSN (2033)	0.59	1.36	1.76
	p-BSN (2844, 2850)	0.59	1.60	1.70
	p-BSN (1930)	0.60	1.25	1.22
	p-BSN (3501)	0.60	1.34	1.82
	p-BSN (3006)	0.61	1.71	1.43
	p-BSN (1154)	0.63	1.59	0.70
	p-BSN (148)	0.64	1.19	0.69
	p-BSN (1468, 1484)	0.65	6.77	5.19
	p-BSN (2850)	0.65	1.55	1.31
	p-BSN (1086, 1089, 1098)	0.67	1.65	2.47
BSN	p-BSN (2055)	0.70	1.76	1.57

	p-BSN (2844)	0.73	1.27	1.31
	p-BSN (105)	0.77	0.26	0.12
	p-BSN (1468)	0.78	2.70	4.48
	p-BSN (1478)	0.79	1.21	1.53
	p-BSN (1469)	0.88	0.95	0.36
	p-BSN (2607)	0.89	0.88	0.78
	p-BSN (1002)	0.91	1.06	0.59
	p-BSN (3367)	0.94	1.20	1.14
	p-BSN (2580)	1.24	1.32	1.08
	p-BSN (717)	1.45	0.83	0.29
	p-BSN (964)	1.59	0.52	0.58
SYN1	p-SYN1 (11)	0.22	1.28	3.21
	p-SYN1 (508)	0.37	1.75	1.54
	p-SYN1 (434)	0.39	11.70	24.67
	p-SYN1 (680)	0.46	1.59	1.52
	p-SYN1 (510)	0.48	0.72	0.99
	p-SYN1 (9)	0.50	0.85	1.12
	p-SYN1 (666)	0.54	1.46	1.41
	p-SYN1 (664, 680)	0.55	1.91	0.40
	p-SYN1 (62)	0.57	1.17	2.47
	p-SYN1 (664)	0.61	1.98	2.69
	p-SYN1 (549)	0.71	0.57	1.30
	p-SYN1 (432, 436)	0.76	1.29	1.47
	p-SYN1 (436)	0.92	1.14	0.82
	p-SYN1 (425, 436)	0.99	1.04	0.93
	p-SYN1 (425)	1.04	1.04	0.66
	p-SYN1 (430)	1.05	0.74	1.55
	p-SYN1 (332)	1.11	1.19	0.92
	p-SYN1 (566)	1.21	0.59	0.48
	p-SYN1 (430, 436)	1.21	0.93	1.21
	p-SYN1 (549, 551)	1.48	0.13	0.16
	p-SYN1 (71)	1.67	1.23	1.58
	p-SYN1 (579)	2.66	1.95	1.14
	p-SYN1 (603)	3.43	0.70	0.23
PICCOLO	p-PICCOLO (1304)	0.51	0.68	1.49
	p-PICCOLO (1841)	0.56	1.52	2.17
	p-PICCOLO (1840)	0.57	2.07	2.47
	p-PICCOLO (814)	0.60	3.72	0.70
	p-PICCOLO (1812)	0.69	1.59	1.61
	p-PICCOLO (1575)	0.74	1.31	0.85
	p-PICCOLO (1820)	0.84	1.34	1.76
	p-PICCOLO (1451)	0.84	1.13	1.03
	p-PICCOLO (3781)	0.85	1.06	0.86
	p-PICCOLO (4343)	0.86	1.17	0.82
	p-PICCOLO (1353)	0.90	1.21	1.06
	p-PICCOLO (4340, 4341)	0.93	1.35	1.87
	p-PICCOLO (1587)	0.93	1.21	0.73
	p-PICCOLO (1772, 1775, 1784)	0.97	0.92	2.01
	p-PICCOLO (1352)	0.97	1.69	0.21
	p-PICCOLO (4150)	1.03	1.02	1.02
	p-PICCOLO (4723)	1.03	1.58	1.28

	p-PICCOLO (3779)	1.09	0.92	0.86
	p-PICCOLO (1772, 1778, 1784)	1.11	0.95	1.42
	p-PICCOLO (4801)	1.19	2.48	0.83
	p-PICCOLO (3614)	1.30	1.22	0.40
	p-PICCOLO (1464)	1.37	1.10	0.76
	p-PICCOLO (1677)	1.58	0.94	0.24
CAMK2B	p-CAMK2B (unk)	0.35	3.21	2.39
	p-CAMK2B (UNK)	0.47	1.65	3.50
	p-CAMK2B (UNK)	0.70	0.90	0.71
	p-CAMK2B (367)	0.75	1.28	1.11
	p-CAMK2B (UNK)	0.86	0.64	1.40
	p-CAMK2B (UNK)	0.86	0.46	0.87
	p-CAMK2B (280)	0.89	1.26	1.35
	p-CAMK2B (UNK)	0.89	2.29	0.67
	p-CAMK2B (UNK)	0.92	0.72	0.75
	p-CAMK2B (UNK)	0.92	1.38	1.43
	p-CAMK2B (UNK)	0.99	1.11	0.85
	p-CAMK2B (UNK)	1.05	0.82	0.76
	p-CAMK2B (UNK)	1.13	0.65	0.39
	p-CAMK2B (UNK)	1.15	0.54	0.60
	p-CAMK2B (277)	1.23	1.73	1.32
	p-CAMK2B (287)	1.25	1.12	1.39
	p-CAMK2B (UNK)	1.77	0.59	0.67
	p-CAMK2B (276)	2.22	1.00	0.84
	p-CAMK2B (UNK)	2.96	0.67	0.82
PLECTIN 1	p-PLECTIN-1 (UNK)	0.63	2.12	2.67
	p-PLECTIN-1 (UNK)	0.80	0.87	1.12
	p-PLECTIN-1 (UNK)	0.89	1.10	1.19
	p-PLECTIN-1 (UNK)	0.90	0.13	0.53
	p-PLECTIN-1 (UNK)	1.05	0.85	1.03
	p-PLECTIN-1 (UNK)	1.14	0.83	1.22
	p-PLECTIN-1 (UNK)	1.17	0.63	0.53
	p-PLECTIN-1 (UNK)	1.20	0.55	0.68
	p-PLECTIN-1 (UNK)	1.21	0.88	1.07
	p-PLECTIN-1 (UNK)	1.25	0.82	1.53
	p-PLECTIN-1 (UNK)	1.52	0.42	0.33
	p-PLECTIN-1 (UNK)	2.90	1.04	4.70
	p-PLECTIN-1 (21)	3.66	0.45	1.83
	p-PLECTIN-1 (UNK)	4.12	0.84	2.27
	p-PLECTIN-1 (UNK)	5.12	0.61	1.42
	p-PLECTIN-1 (UNK)	6.06	0.24	0.88
	p-PLECTIN-1 (UNK)	13.61	0.24	1.27
NDRG1	p-NDRG1 (375)	0.52	1.01	0.87
	p-NDRG1 (330, 333)	0.74	0.73	0.80
	p-NDRG1 (333, 336)	0.90	0.42	0.89
	p-NDRG1 (367)	1.05	0.91	1.01
	p-NDRG1 (333)	1.15	0.55	0.48
	p-NDRG1 (330)	1.26	0.52	0.51
	p-NDRG1 (332, 333)	1.30	0.51	0.40
	p-NDRG1 (319)	1.53	0.47	0.69
	p-NDRG1 (332)	1.53	0.43	0.43

	p-NDRG1 (328, 332, 336)	1.57	0.31	0.31
	p-NDRG1 (366)	1.63	0.78	0.92
	p-NDRG1 (328, 330)	2.02	0.67	0.87
	p-NDRG1 (342)	2.32	1.21	1.17
	p-NDRG1 (330, 333, 336)	2.96	0.37	1.75
	p-NDRG1 (362, 364)	3.23	0.52	0.45
RIMS1	p-RIMS1 (UNK)	0.33	2.18	1.79
	p-RIMS1 (UNK)	0.33	2.96	3.68
	p-RIMS1 (UNK)	0.50	1.55	0.10
	p-RIMS1 (UNK)	0.54	1.93	1.03
	p-RIMS1 (UNK)	0.57	0.89	0.66
	p-RIMS1 (UNK)	0.60	1.28	1.57
	p-RIMS1 (UNK)	0.61	1.15	1.87
	p-RIMS1 (UNK)	0.74	1.13	0.83
	p-RIMS1 (UNK)	0.78	1.56	1.25
	p-RIMS1 (UNK)	0.83	1.36	0.94
	p-RIMS1 (UNK)	0.86	0.61	0.52
	p-RIMS1 (UNK)	0.89	1.25	1.35
	p-RIMS1 (UNK)	1.06	0.99	1.43
	p-RIMS1 (UNK)	1.55	1.34	2.88
RAB11FIP5	p-RAB11FIP5 (606;483)	0.23	3.33	3.23
	p-RAB11FIP5 (612;489)	0.37	2.72	2.68
	p-RAB11FIP5 (UNK)	0.49	1.15	1.54
	p-RAB11FIP5 (UNK)	0.50	0.93	1.55
	p-RAB11FIP5 (533)	0.51	1.46	1.13
	p-RAB11FIP5 (UNK)	0.55	1.27	1.16
	p-RAB11FIP5 (419;296)	0.70	2.07	2.51
	p-RAB11FIP5 (UNK)	0.78	0.99	0.86
	p-RAB11FIP5 (430;307)	1.00	1.03	1.20
	p-RAB11FIP5 (299;176)	1.40	1.15	2.10
	p-RAB11FIP5 (330;207)	2.66	0.38	0.15
PARALEMMIN	p-PARALEMMIN (145)	0.39	1.71	2.92
	p-PARALEMMIN (141)	0.45	1.84	4.53
	p-PARALEMMIN (363)	0.46	1.29	1.28
	p-PARALEMMIN (157)	0.48	6.33	3.38
	p-PARALEMMIN (116, 124)	0.56	1.61	1.48
	p-PARALEMMIN (153, 157)	0.72	1.79	1.38
	p-PARALEMMIN (153)	0.91	1.32	1.00
	p-PARALEMMIN (124)	0.99	1.04	0.86
	p-PARALEMMIN (122)	1.01	1.04	0.16
	p-PARALEMMIN (265)	1.01	1.30	1.15
	p-PARALEMMIN (123, 124)	1.18	0.90	0.99
DMTN	p-DMTN (267)	0.89	0.85	0.51
	p-DMTN (UNK)	0.90	1.27	1.35
	p-DMTN (87)	0.95	0.78	4.28
	p-DMTN (UNK)	0.95	0.78	0.62
	p-DMTN (269)	0.99	0.85	0.55
	p-DMTN (UNK)	1.04	1.00	0.99
	p-DMTN (289)	1.13	1.10	1.04
	p-DMTN (96)	1.16	1.42	1.06
	p-DMTN (92)	1.35	1.30	1.35

	p-DMTN (22, 26)	1.46	2.25	1.14
	p-DMTN (226)	2.34	0.61	0.90
CACNA1A	p-CACNA1A (752)	0.42	1.46	2.17
	p-CACNA1A (UNK)	0.46	0.57	0.46
	p-CACNA1A (UNK)	0.49	1.76	0.49
	p-CACNA1A (2071)	0.62	0.92	1.05
	p-CACNA1A (UNK)	0.78	3.92	1.84
	p-CACNA1A (792)	1.01	0.15	0.13
	p-CACNA1A (2028)	1.06	1.10	0.75
	p-CACNA1A (UNK)	1.39	1.47	1.24
	p-CACNA1A (UNK)	1.40	0.82	0.76
	p-CACNA1A (2068)	1.45	0.36	0.08
ANK3	p-ANK3 iso2 (1854)	0.11	3.89	10.94
	p-ANK3 iso2 (631;606)	0.49	1.79	2.34
	p-ANK3 iso2 (2457)	0.64	1.15	1.34
	p-ANK3 iso2 (2102)	0.85	0.42	0.79
	p-ANK3 iso2 (1458)	0.88	1.36	2.31
	p-ANK3 iso2 (1984)	0.97	1.54	1.19
	p-ANK3 iso2 (2247)	1.84	0.40	0.72
	p-ANK3 iso2 (869, 875)	2.47	0.33	0.44
	p-ANK3 iso2 (875)	3.06	0.59	0.54
NF1	p-NF1 (866)	0.83	1.25	1.07
	p-NF1 (2496)	0.89	0.90	1.14
	p-NF1 (2783)	0.97	1.15	0.42
	p-NF1 (2524)	1.00	1.05	1.22
	p-NF1 (2495)	1.07	0.90	0.84
	p-NF1 (2580)	1.20	0.57	0.82
	p-NF1 (2578)	1.21	0.56	1.08
	p-NF1 (2169)	1.85	0.77	0.49
MGLUR5	p-MGLUR5 (860)	0.09	7.29	6.76
	p-MGLUR5 (901;933)	0.18	8.26	12.33
	p-MGLUR5 (839)	0.35	1.65	0.10
	p-MGLUR5 (1141, 1185)	0.38	1.41	1.60
	p-MGLUR5 (1152;1185)	0.63	1.04	0.63
	p-MGLUR5 (1016)	0.65	1.75	1.79
	p-MGLUR5 (1170;1202)	0.87	1.67	1.25
SPARCL1	p-SPARCL1 (340)	0.49	3.03	4.68
	p-SPARCL1 (68)	0.74	1.19	0.87
	p-SPARCL1 (68, 76)	0.81	1.32	1.10
	p-SPARCL1 (76)	0.82	1.15	0.80
	p-SPARCL1 (333, 340)	0.95	0.99	0.67
	p-SPARCL1 (68, 77)	1.02	1.23	0.96
	p-SPARCL1 (151)	1.22	1.04	0.30
NCAM-L1	p-NCAM-L1 iso2 (1173, 1180)	0.91	0.37	1.70
	p-NCAM-L1 iso2 (1175;1180)	1.02	1.20	0.63
	p-NCAM-L1 iso2 (1173;1178)	1.06	1.01	0.58
	p-NCAM-L1 iso2 (1178;1183)	1.07	0.92	1.60
	p-NCAM-L1 iso2 (1160, 1174)	1.10	0.85	1.14
	p-NCAM-L1 iso2 (UNK)	1.21	0.93	0.77
	p-NCAM-L1 iso2 (1173, 1183)	1.66	0.86	0.85
	p-ITGB4 (1405)	1.12	0.82	1.31

ITGB4	p-ITGB4 (1121)	1.37	0.68	1.36
	p-ITGB4 (1366)	2.72	0.56	2.34
	p-ITGB4 (1776)	3.61	0.34	1.36
	p-ITGB4 (1389)	9.06	0.73	2.90
SAPAP3	p-SAPAP3 (750)	0.16	6.44	0.49
	p-SAPAP3 (185)	0.59	2.03	2.34
	p-SAPAP3 (58)	0.80	1.13	0.22
	p-SAPAP3 (712)	1.14	1.30	1.06
	p-SAPAP3 (965)	3.79	1.72	0.30
UNC13C	p-UNC13C (450)	0.65	1.79	1.21
	p-UNC13C (89)	0.68	1.33	1.19
	p-UNC13C (570)	0.93	1.94	1.36
	p-UNC13C (883)	1.37	0.69	0.85
	p-UNC13C (537)	6.28	0.29	0.12
RABPHILIN 3A	p-RABPHILIN 3A (683)	0.71	1.64	1.66
	p-RABPHILIN 3A (281;284)	0.94	1.05	1.50
	p-RABPHILIN 3A (682)	0.96	1.31	1.42
	p-RABPHILIN 3A (259)	1.18	0.61	0.63
	p-RABPHILIN 3A (262)	1.41	0.72	0.41
FLNA	p-FLNA (2172;2180)	0.90	1.07	1.57
	p-FLNA (UNK)	1.08	0.69	1.35
	p-FLNA (968)	1.65	0.71	0.91
	p-FLNA (1459)	2.30	0.34	0.29
	p-FLNA (2144;2152)	2.40	0.50	0.69
PTPRN2	p-PTPRN2 (687)	0.79	1.39	1.46
	p-PTPRN2 (425)	1.26	1.16	0.81
	p-PTPRN2 (330)	1.47	0.85	0.78
	p-PTPRN2 (UNK)	2.11	0.75	0.94
PPFIA3	p-PPFIA3 (1162;1153)	0.16	3.32	8.32
	p-PPFIA3 (142)	0.85	1.01	1.11
	p-PPFIA3 (667)	0.85	2.26	0.41
	p-PPFIA3 (712)	1.27	0.59	1.00
RIMS2	p-RIMS2 (UNK)	0.48	2.22	4.02
	p-RIMS2 (UNK)	0.66	1.19	1.05
	p-RIMS2 (UNK)	0.78	0.74	0.23
	p-RIMS2 (UNK)	1.46	1.42	1.65
PRR14	p-PRR14 (80)	0.73	0.83	0.73
	p-PRR14 (164;71)	0.86	1.40	1.06
	p-PRR14 (78)	1.06	1.63	1.24
	p-PRR14 (158;65)	1.32	1.01	1.09
SNTB1	p-SNTB1 (88)	1.68	0.50	0.47
	p-SNTB1 (215)	1.55	0.65	0.48
	p-SNTB1 (390)	1.50	0.81	0.76
	p-SNTB1 (215, 220)	1.09	0.41	0.95
CACNB4	p-CACNB4 (UNK)	0.44	1.78	1.83
	p-CACNB4 (377)	0.88	1.38	0.72
	p-CACNB4 (UNK)	2.53	0.74	0.58
DRP2	p-DRP2 (UNK)	1.47	0.74	1.05
	p-DRP2 (UNK)	2.56	0.46	0.53
	p-DRP2 (UNK)	6.34	0.44	1.03
	p-SPTBN2 (2254)	0.65	1.27	1.34

SPTBN2	p-SPTBN2 (2199)	0.90	0.97	0.97
	p-SPTBN2 (2200;2207)	1.10	0.90	0.79
GJC3	GJC3 (244)	4.52	0.22	1.62
	GJC3 (233)	1.86	0.97	1.12
	GJC3 (238)	1.19	0.74	1.13
PTPRN	p-PTPRN (UNK)	1.51	1.16	1.57
	p-PTPRN (UNK)	1.59	1.58	1.48
	p-PTPRN (311)	1.67	0.90	0.62
NEURABIN 1	p-NEURABIN 1 (192)	0.32	0.43	2.94
	p-NEURABIN 1 (UNK)	0.62	0.87	1.34
	p-NEURABIN 1 (372)	0.87	1.07	0.78
MYO5A	p-MYO5A (600)	1.07	0.96	1.08
	p-MYO5A (1624)	1.56	0.61	0.13
	p-MYO5A (1115)	1.77	0.71	0.77
DBNL	p-DBNL (24)	0.92	0.93	0.90
	p-DBNL (311)	1.03	0.39	0.55
	p-DBNL (291)	1.65	0.49	0.73
TALIN 1	p-TALIN 1 (1260;1277)	1.47	0.52	0.67
	p-TALIN 1 (425)	2.24	0.74	1.07
PSD-95	p-PSD-95 (UNK)	0.77	1.16	1.44
	p-PSD-95 (415)	1.31	1.03	0.60
TNC	p-TNC (72)	3.56	0.47	0.07
FGF13	p-FGF13 (UNK)	0.87	1.40	1.26
CHAT	p-CHAT (365)	1.61	0.41	0.59
RAB3A	p-RAB3A (63)	1.69	0.51	0.70
DAG1	p-DAG1 (788)	8.36	0.30	0.20
PXN	p-PXN (UNK)	1.50	0.51	0.67
APOE	p-APOE (139)	2.50	0.60	0.64
CADM1	p-CADM1 (468)	1.32	0.77	0.93
CTNNA1	p-CTNNA1 (643)	1.18	0.70	0.90
PDK1	p-PDK1 (244;217)	0.89	1.20	0.90
HAPLN4	HAPLN4 (195)	0.86	1.63	0.09
MYO1E	p-MYO1E (1001)	0.86	2.06	0.28
WAVE1	p-WAVE1 (310)	0.49	0.72	1.36
CNTNAP	p-CNTNAP (1380)	1.12	1.14	1.00
ERC2	p-ERC2 (910)	0.78	0.86	0.59
MMP15	p-MMP15 (587)	0.53	0.65	0.39
PAFAH1B1	p-PAFAH1B1 (56)	0.91	0.92	0.61
FBN1	p-FBN1 (2710)	1.60	1.32	1.96
nNOS	p-nNOS (858)	1.19	1.40	0.95

Numbers in parenthesis indicate phosphorylated residues. Comma separation indicates multiple phosphorylation. Semicolon separation indicates possible residue location
